# Scalable Implementation of Mean-Field and Correlation Methods Based on Lie-Algebraic Similarity Transformation of Spin Hamiltonians in the Jordan–Wigner Representation

**DOI:** 10.1021/acs.jctc.5c01412

**Published:** 2025-10-31

**Authors:** Shadan Ghassemi Tabrizi, Thomas M. Henderson, Thomas D. Kühne, Gustavo E. Scuseria

**Affiliations:** † Computational System Sciences, Technische Universität Dresden, 01187 Dresden, Germany; ‡ Center for Advanced Systems Understanding (CASUS), Am Untermarkt 20, 02826 Görlitz, Germany; § Department of Physics and Astronomy, 3990 Rice University, Houston, Texas 77005-1892, United States; ∥ Department of Chemistry, Rice University, Houston, Texas 77005-1892, United States

## Abstract

Recent work has highlighted that the strong correlation inherent in spin Hamiltonians can be effectively reduced by mapping spins to Fermions via the Jordan–Wigner transformation (JW). The Hartree–Fock method is straightforward in the Fermionic domain and may provide a reasonable approximation to the ground state. Correlation with respect to the Fermionic mean field can be recovered based on Lie-algebraic similarity transformation (LAST) with two-body correlators. Specifically, a unitary LAST variant eliminates the dependence on site ordering, while a nonunitary LAST yields size-extensive correlation energies. Whereas the first recent demonstration of such methods was restricted to small spin systems, we present efficient implementations using analytical gradients for the optimization with respect to the mean-field reference and the LAST parameters, thereby enabling the treatment of larger clusters, including systems with local spins $s>\frac{1}{2}$ .

## Graphical abstract

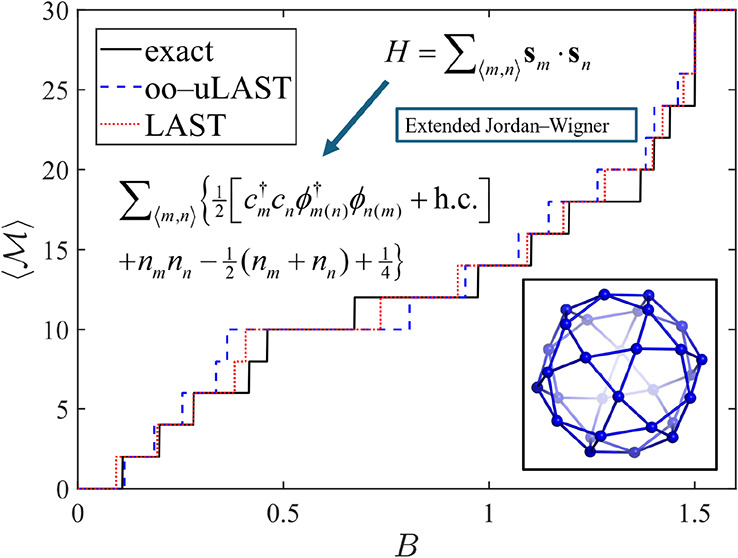

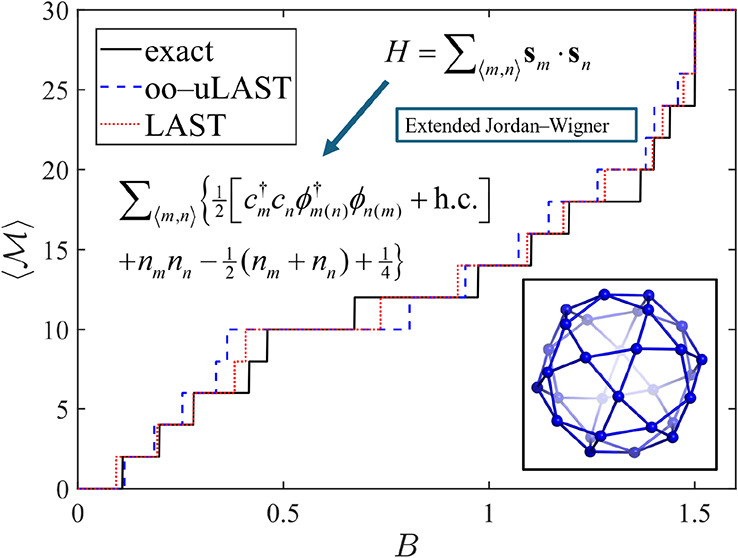

## 1 Introduction

The physics of exchange-coupled systems is frequently described in terms of interacting spin centers.^1–3^ As an example, the isotropic Heisenberg model, *H* = Σ_*m*<*n*_ *J*_*mn*_ **s**_*m*_·**s**_*n*_, containing pairwise scalar couplings between site spins **s**_*m*_ = (*s*_*m*_^*x*^, *s*_*m*_^*y*^, *s*_*m*_^*z*^), can be used to represent the properties of multinuclear transition-metal complexes or magnetic solids. Because the state-space dimension grows rapidly with the number of sites, approximations are required^4–6^ to obtain low-lying states (for interpreting spectroscopic data) or to compute thermal averages (e.g., temperature-dependent magnetic susceptibilities). Mean-field techniques belong to the less computationally demanding end of the spectrum of methods. Among these is projected Hartree–Fock theory^7^ (PHF), where a three-dimensional spin configuration^8^ that breaks spin and point-group symmetry is optimized with respect to projectors that restore these symmetries, thereby affording a state with definite quantum numbers. The loss of accuracy with increasing system size (PHF is not size-extensive) can be prevented by constructing a cluster basis^9,10^ or by including additional configurations in the broken-symmetry reference.^11^ Note that several other correlated methods widely used in quantum chemistry, such as many-body perturbation theory, coupled-cluster theory, or configuration interaction, can be applied to spin systems as well,^12,13^ preferably based on a cluster mean-field reference.^14^ Although these approaches can adopt certain technical features of quantum-chemical implementations, they still fundamentally work in the spin representation, where the simplest mean-field state (a direct product of local spins) provides a poor approximation to the ground state.

In contrast, mapping spins to Fermions via the Jordan–Wigner transformation^15^ (JW) and other techniques^16^ reduces strong correlations at the mean-field level.^16,17^ Consequently, significantly more correlation is captured compared with an HF approach in the spin picture. Applications are not limited to one-dimensional systems with nearest-neighbor coupling (JW strings vanish in chains and reduce to a particle-number-dependent sign factor in rings^18^): on general connectivity graphs, e.g., two- or three-dimensional lattices, the nonlocal JW strings can be evaluated as simple Thouless rotations^19^ of a Slater determinant. Therefore, no mean-field decoupling of many-body terms arising from string operators is required.^17^ In any case, such a decoupling is only practically feasible in special cases where a formulation of strings (eq 4) as Π_*q*<*p*_ (1–*i*π*n*_*q*_) does not generate three- or higher-body terms in the Hamiltonian, see, e.g., refs 20–22 . Moreover, it was recently demonstrated that a site-order independent formulation can be derived by generalizing the transformation toward extended JW (EJW^23,24^ ) and treating the additional degrees of freedom afforded by EJW as variational parameters. This corresponds to a unitary Lie-algebraic similarity transformation (uLAST) approach^25^ and becomes orbital-optimized uLAST (oo-uLAST) when combined with an optimization of the Slater determinant,^25^ which provides an attractive foundation for correlation methods like nonunitary LAST.^25,26^ Throughout this work, oo-uLAST is a size-consistent variational method and therefore obeys the Ritz variational principle within a fixed Fermion-number sector, while the subsequent nonunitary LAST step (in the following simply referred to as LAST) is not variational and may occasionally yield energies below the exact ground state.

Here, we present efficient formulations for gradient-based HF, oo-uLAST, and subsequent LAST in JW-transformed spin Hamiltonians. This enables the treatment of larger clusters than those previously feasible by these methods. In the following section, we briefly revisit the standard JW transformation and its extended variant. As noted, regarding the EJW phase angles as variational parameters—motivated by the fact that EJW can be derived from uLAST—eliminates the dependence on site ordering and can, in certain cases, when HF orbitals become complex, provide additional correlation.^25^ We derive analytical gradients for oo-uLAST and the analytical Jacobian for the equations determining the LAST amplitudes. The EJW angles (the uLAST parameters) and the reference determinant (the occupied orbitals) are optimized simultaneously in oo-uLAST, and LAST is employed to obtain a size-extensive correlation on top of an oo-uLAST solution. In Section 3, oo-uLAST and LAST are benchmarked against exact-diagonalization (ED) data for larger systems than previously studied in the frame of an initial implementation,^25^ which operated in the full configuration-interaction (FCI) basis. Local spins $s>\frac{1}{2}$ are treated by decomposing them into spin-1/2 degrees of freedom, which are then Fermionized through EJW. Finally, we discuss how these methods can serve as a basis for recovering additional correlation.

## 2 Theory

The JW-transformation maps spins to Fermions or vice versa. As an example, Fermionic Hamiltonians are expressed in terms of qubit operators for quantum-computing applications. Here, we use the reverse direction

1 $$s_{p}^{+}=c_{p}^{†}\phi_{p}^{†}$$

2 $$s_{p}^{-}=c_{p}\phi_{p}$$

3 $$s_{p}^{z}=n_{p}-\frac{1}{2}$$

because the Fermionic domain typically mitigates strong correlation compared to the spin representation, allowing a mean-field treatment based on a single Slater determinant to provide a qualitatively reasonable approximation of the ground state.^25^ In eqs 1 and 2, *s*_*p*_^+^ = *s*_*p*_^*x*^ + *is*_*p*_^*y*^ and *s*_*p*_^–^ = *s*_*p*_^*x*^ − *is*_*p*_^*y*^ are spin ladder operators at site *p*, and *c*_*p*_^†^ (*c*_*p*_) are Fermion creation (annihilation) operators. Equation 4 specifies the standard JW string

4 $$\phi_{p}^{†}=\prod_{q<p}e^{i\pi n_{q}}$$

Because the total Fermion number, Σ_*p*_ *n*_*p*_ = Σ_*p*_ *c*_*p*_^†^ *c*_*p*_, corresponds (up to a constant) to the *z*-component of the total spin, *S*_*z*_ = Σ_*p*_ *s*_*p*_^*z*^, cf. eq 3, the HF approximation is limited to Hamiltonians with *S*_*z*_ symmetry, like the *XXZ* or *J*_1_–*J*_2_ models studied here. While fixing the total magnetization *M* (the *S*_*z*_ eigenvalue) is thereby straightforward, a general adaptation to SU(2) or point-group symmetry, which is straightforward in the spin representation,^8^ becomes nontrivial, because spin rotations and lattice symmetries map to nonlocal operators under the JW transformation. In the fully anisotropic case, where no spin component is conserved and thus number symmetry and number parity are broken, more general mean-field methods would be needed.^27,28^

EJW represents a generalization of eq 4, which still ensures the correct commutation (anticommutation) relations for spins (Fermions) by introducing the real parameters θ_*pq*_ subject to the constraints |θ_*pq*_ – θ_*qp*_| = π, θ_*pp*_ = 0, see eq 5

5 $$\phi_{p}^{†}=e^{i\sum_{q}\theta_{\mathrm{pq}}n_{q}}$$

Without loss of generality, we may set θ_*qp*_ = θ_*pq*_ + π for *p* < *q*. The EJW strings are unitary and commute with their associated creation and annihilation operators, e.g., [*c*_*p*_, ϕ_*p*_] = 0. However, in contrast to standard JW, EJW strings are generally not Hermitian, i.e., ϕ_*p*_^†^ ≠ ϕ_*p*_. Equation 6 shows the transformation of the *x*- and *y*-coupling contributions for a pair ⟨*m*,*n*⟩.

6 $$s_{m}^{x}s_{n}^{x}+s_{m}^{y}s_{n}^{y}=\frac{1}{2}\left(s_{m}^{+}s_{n}^{-}+s_{n}^{+}s_{m}^{-}\right)=\frac{1}{2}\left(c_{m}^{†}\phi_{m}^{†}c_{n}\phi_{n}+c_{n}^{†}\phi_{n}^{†}c_{m}\phi_{m}\right)$$

To treat the strings as Thouless rotations^19^ of a Slater determinant [see eq 43 in the Appendix A1], they are shifted to the right according to eq 7

7 $$\phi_{m}^{†}c_{n}=e^{i\sum_{q}\theta_{\mathrm{mq}}n_{q}}c_{n}=c_{n}e^{i\sum_{q\neq n}\theta_{\mathrm{mq}}n_{q}}$$

yielding eq 8

8 $$s_{m}^{x}s_{n}^{x}+s_{m}^{y}s_{n}^{y}=\frac{1}{2}\left[c_{m}^{†}c_{n}\phi_{m\left(n\right)}^{†}\phi_{n\left(m\right)}+h\mathrm{.c}.\right]$$

where ϕ_*m*(*n*)_^†^ ≡ *e* ^*i*Σ_*q*≠*n*_θ_*mq*_ *n*_*q*_^ is a reduced string. (In the first term of eq 8, ϕ_*n*(*m*)_ could be identically replaced by ϕ_*n*_, but we choose the present more symmetrical notation.) Equation 9 formulates the *z*-coupling in normal order.

9 $$s_{m}^{z}s_{n}^{z}=n_{m}n_{n}-\frac{1}{2}\left(n_{m}+n_{n}\right)+\frac{1}{4}=c_{m}^{†}c_{n}^{†}c_{n}c_{m}-\frac{1}{2}\left(n_{m}+n_{n}\right)+\frac{1}{4}$$

Thus, in either standard JW or EJW, the spin Hamiltonian (for the sake of a slightly simpler notation, we focus here on the Heisenberg model instead of the *XXZ* model) can be faithfully mapped onto the Fermionic Hamiltonian of eq 10

10 $$H=\sum_{m<n}J_{\mathrm{mn}}\left\{\frac{1}{2}\left[c_{m}^{†}c_{n}\phi_{m\left(n\right)}^{†}\phi_{n\left(m\right)}+h\mathrm{.c}.\right]+n_{m}n_{n}-\frac{1}{2}\left(n_{m}+n_{n}\right)+\frac{1}{4}\right\}$$

Consistency with the convention of fully antisymmetrized two-particle integrals in a second-quantized Hamiltonian^29^ is established through eq 11

11 $$c_{m}^{†}c_{n}^{†}c_{n}c_{m}=\frac{1}{4}\left(c_{m}^{†}c_{n}^{†}c_{n}c_{m}+c_{n}^{†}c_{m}^{†}c_{m}c_{n}-c_{m}^{†}c_{n}^{†}c_{m}c_{n}-c_{n}^{†}c_{m}^{†}c_{n}c_{m}\right)$$

This allows the *z*-coupling part, *H*_*z*_, to be written in a standard form, eq 12, with appropriately chosen values for the one- and two-particle integrals, *t*_*kl*_ and [*kn*|*lm*], respectively, and $E_{\mathrm{const}}=\frac{1}{4}\sum_{m<n}J_{\mathrm{mn}}$ . We adopt this formulation in the derivations provided in the Appendix.

12 $$H_{z}\equiv \sum_{m<n}J_{\mathrm{mn}}s_{m}^{z}s_{n}^{z}=E_{\mathrm{const}}+\sum_{\mathrm{kl}}t_{\mathrm{kl}}c_{k}^{†}c_{l}+\frac{1}{2}\sum_{\mathrm{klmn}}\left[\mathrm{kn}|\mathrm{lm}\right]c_{k}^{†}c_{l}^{†}c_{m}c_{n}$$

### 2.1 HF and oo-uLAST

The HF method consists of variationally optimizing a Slater determinant |Φ⟩. In quantum-chemical terminology, *a*_*o*_^†^ acting on the vacuum |0⟩ in eq 13 creates a Fermion in a molecular orbital (indices *o* and *v* will be used for occupied and virtual MOs, respectively)

13 $$\left|\Phi \right\rangle =\left(\prod_{o\in \mathrm{occ}}a_{o}^{†}\right)\left|0\right\rangle$$

However, for fixed strings (e.g., standard JW), the HF energy depends on the site numbering. This might appear to be problematic for nonlinear lattices only, but even in *XXZ* rings or chains, depending on the Δ parameter (see eq 29), a successive ordering may not be optimal in terms of the HF energy.^25^ Treating EJW angles θ_*pq*_ as optimization parameters corresponds to a special form of uLAST and makes the ansatz independent of the site numbering.^25^ The simplest unitary or nonunitary LAST variants employ a two-body correlator,^26^ eq 14

14 $$\gamma_{2}=\frac{1}{2}\sum_{p,q}\gamma_{\mathrm{pq}}n_{p}n_{q}$$

Only the γ_*pq*_ parameters with *p* < *q* are independent. When setting γ_*pq*_ = *i*Θ_*pq*_, with a real symmetric **Θ**, e^γ_2_^ becomes unitary. The EJW-transformed Hamiltonian, with θ_*pq*_ = Θ_*pq*_ for *p* < *q* and θ_*pq*_ = Θ_*pq*_ + π for *p* > *q*, is obtained from the standard JW form (cf. eq 4) through the unitary transformation of eq 15

15 $$H_{\mathrm{EJW}}=e^{-\gamma_{2}}H_{\mathrm{JW}}e^{\gamma_{2}}$$

Henceforth, it will be clear from the context whether *H* represents *H*_EJW_ or *H*_JW_ (an explicit differentiation is usually not necessary). In the following two sections and in Appendix (A1 and A2), we discuss the simultaneous optimization (using analytical gradients) of the Slater determinant and the angles θ_*pq*_, which constitutes the oo-uLAST method.

For a given *H*, the optimization of |Φ⟩ corresponds to a HF procedure. |Φ⟩ is defined in terms of a Thouless rotation acting on an initial guess |Φ^0^⟩ = Π_*o*_(*a*_*o*_^0^)^†^|0⟩, eq 16

16 $$\left|\Phi \right\rangle =N\left(Z\right)e^{Z}\left|\Phi^{0}\right\rangle$$

where *N*(*Z*) is a normalization constant, and *Z* is defined in eq 17

17 $$Z=\sum_{v\in \mathrm{virt}}\sum_{o\in \mathrm{occ}}Z_{\mathrm{vo}}a_{v}^{†}a_{o}$$

The minimization of the HF energy, eq 18

18 $$E=\frac{\langle \Phi^{0}|e^{Z^{†}}He^{Z}\left|\Phi^{0}\right\rangle }{\langle \Phi^{0}|e^{Z^{†}}e^{Z}\left|\Phi^{0}\right\rangle }$$

with respect to the amplitudes *Z*_*vo*_ is detailed in Appendix A1, and the uLAST optimization of the angles θ_*pq*_ is explained in Appendix A2.

### 2.2 LAST

As in ref ^25^, we apply nonunitary LAST to capture additional correlation energy for an oo-uLAST solution. We derive and implement the analytic Jacobian for the LAST amplitudes, enabling an efficient optimization procedure. The two-body correlator is parametrized by a real symmetric matrix **α**, with α_*pp*_ = 0, cf. eq 19

19 $$\alpha_{2}=-\frac{1}{2}\sum_{p,q}\alpha_{\mathrm{pq}}n_{p}n_{q}$$

The respective transformations of creation and annihilation operators are given in eqs 20 and 21.^25,26^ Note that *c̅*_*p*_^†^ and *c̅*_*p*_ are not Hermitian conjugates to each other.

20 $${\mathrm{c̅}}_{p}^{†}\equiv e^{-\alpha_{2}}c_{p}^{†}e^{\alpha_{2}}=c_{p}^{†}e^{\sum_{q}\alpha_{\mathrm{pq}}n_{q}}$$

21 $${\mathrm{c̅}}_{p}\equiv e^{-\alpha_{2}}c_{p}e^{\alpha_{2}}=c_{p}e^{-\sum_{q}\alpha_{\mathrm{pq}}n_{q}}$$

The transformed Hamiltonian of eq 22

22 $$\mathrm{H̅}=e^{-\alpha_{2}}H_{\mathrm{EJW}}e^{\alpha_{2}}$$

is still given by eq 10 above, but now reduced strings take the form of eq 23

23 $$\phi_{m\left(n\right)}^{†}=e^{\sum_{q\neq n}\left(\alpha_{\mathrm{mq}}+i\theta_{\mathrm{mq}}\right)n_{q}}$$

Using LAST on top of oo-uLAST means that the mean-field state |Φ⟩ and uLAST parameters **θ** remain fixed, and **α** is optimized to solve the system of eq 24 for the residuals *R*_*pq*_ of all independent index pairs (*p* < *q*)^26^

24 $$R_{\mathrm{pq}}\equiv \left\langle \Phi \right|n_{p}n_{q}\left(\mathrm{H̅}-E\right)\left|\Phi \right\rangle =0$$

where the energy is given in eq 25

25 $$E=\frac{\left\langle \Phi \right|\mathrm{H̅}\left|\Phi \right\rangle }{\left\langle \Phi \right|\Phi \rangle }$$

It is useful to pass the Jacobian, defined in eq 26, to the numeric solver (in our Matlab program, we use the fsolve function).

26 $$J_{\mathrm{pq}}^{\mathrm{rs}}\equiv \frac{\partial R_{\mathrm{pq}}}{\partial \alpha_{\mathrm{rs}}}$$

Equations for the computation of the energy, the residuals, and the Jacobian are provided in Appendix A3.

### 2.3 Spin-1/2 Decomposition for *s* > 1

A well-known example of representing on-site spins $s>\frac{1}{2}$ by auxiliary spin-1/2 degrees of freedom (qubits) is the Affleck–Kennedy–Lieb–Tasaki^30^ (AKLT) construction for the *s* = 1 chain: each physical spin is modeled by two qubits, singlet bonds are formed between qubits on neighboring sites, and sites are finally projected onto their local triplet subspace. A valence-bond-solid wave function is the exact ground state of the AKLT parent Hamiltonian with bilinear–biquadratic couplings and, in modern terms, corresponds to a matrix-product state of bond dimension two. However, we mention this construction only to motivate the auxiliary-qubit picture used below (our decomposition does not assume the AKLT parent Hamiltonian, nor do we enforce any local-spin projection). As other examples of applications of spin-1/2 decompositions, we mention a technique to constrain the PHF mean-field reference to represent a spin-coherent product state,^8^ and the selection of spin configurations that form a linearly independent and complete set of spin eigenfunctions upon projection onto a total-spin subspace.^31^ Here, we introduce spin-1/2 particles, eq 27

27 $$s_{p}\to \sum_{a=1}^{2s_{p}}\kappa_{p,a}$$

to bring systems with local spins $s_{p}>\frac{1}{2}$ into the Fermionic mean-field framework by applying a JW- or EJW-transformation to the qubits **κ**_*p*,*a*_. We are aware of only a few previous works where a similar decomposition combined with a JW mapping has been used, e.g., anisotropy-dependent phase diagrams and the Haldane gap in *s* = 1 chains were studied in refs 32,33 . To the best of our knowledge, there are no previous applications of this strategy for *s* > 1. Even for an *s* = 1 chain, the JW mapping already carries nonlocal strings (in contrast to the $s=\frac{1}{2}$ chain). Then, the coupling between most distant qubit pairs, i.e., the first and the second qubits of sites *m* and *m* + 1, **κ**_*m*,1_ and **κ**_*m*+1,2_, respectively (see eq 28), generates six-body terms when expanding string factors as *e* ^*i*π*n*_*p*_^ = 1–*i*π*n*_*p*_. (In a conventional successive numbering of spin-1/2 particles, the *x*,*y*-coupling between **κ**_*m*,1_ and **κ**_*m*+1,2_ involves a string *e* ^*i*π(*n*_*m*,2_+*n*_*m*+1,1_)^. The *x*,*y*-coupling thus contributes *c*_*m*,1_^†^ *c*_*m*+1,2_ *n*_*m*,2_ *n*_*m*+1,1_, among other terms.) Therefore, unless one evaluates the strings exactly as Thouless rotations,^17^ as we do here, a mean-field decoupling would generally become unwieldy for $s>\frac{1}{2}$ , even in one-dimensional systems.

In the enlarged Hilbert space of *H̃*, eq 28

28 $$H=\sum_{m<n}J_{\mathrm{mn}}s_{m}\cdot s_{n}\to \mathrm{H̃}=\sum_{m<n}J_{\mathrm{mn}}\sum_{a,b}\kappa_{m,a}\cdot \kappa_{n,b}$$

**κ**_*p*,*a*_ can be coupled into on-site spin values that are smaller than *s*_*p*_. Enforcing the physical sector of maximal local spins would require full symmetrization of the auxiliaries at every site, which is impractical. Besides the rapidly increasing length of the respective linear combinations with the number of sites, permutations of spin-1/2 particles generally do not correspond to simple orbital permutations in the JW picture (this contrasts with the spin representation used in PHF,^8^ where symmetry projection is achieved by Thouless rotations of orbitals). However, contamination by unphysical states with lower on-site spins does not affect the variational principle for the ground state because the latter has maximal local spins, as proven in Appendix A4. Consequently, the ground state – and, indeed, the ground state in each magnetization sector of the *XXZ* model – is fully embedded in the correct state space, i.e., any trial wave function in *H̃* is variationally consistent with *H*.

Although not considered here, we would like to mention another Fermionization scheme for $s>\frac{1}{2}$ proposed by Batista and Ortiz.^34^ It associates the 2*s* + 1 states of each site with the occupation state of an orbital, which may be empty or host a Fermion with 2*s* different flavors. However, in contrast to the auxiliary spin-1/2 decomposition, unphysical contributions (corresponding to multiply occupied sites) may affect the validity of the variational principle for the ground state and would have to be excluded.

Lastly, the constraint-free mapping for spins with multiplicities (2*s* + 1) = 2^*n*^ suggested by Dobrov^35^ is based on the equivalence of the dimensions of spin and Fermion spaces. While it reduces to standard JW for $s=\frac{1}{2}$ , several difficulties occur for $s>\frac{1}{2}$ . In this scheme, even the isotropic Heisenberg model breaks Fermion-number parity, which would require specialized mean-field approaches^27,28^ involving Hartree–Fock–Bogoliubov^36^ (HFB) states, where matrix elements between HFB states may have to be computed in a numerically robust formulation.^37^ Another hurdle for practical applications is high-rank Fermionic terms, e.g., pairwise couplings between $s=\frac{7}{2}$ sites afford five-body interactions (not counting JW strings).

## 3 Results

To explore systems beyond the size accessible to an initial testing implementation,^25^ we consider isotropic rings with up to 30 sites. We also explore the performance of JW-HF, oo-uLAST, and LAST for the *XXZ* model

29 $$H=\sum_{\left\langle m,n\right\rangle }\left(s_{m}^{x}s_{n}^{x}+s_{m}^{y}s_{n}^{y}+\Delta s_{m}^{z}s_{n}^{z}\right)$$

where the sum in eq 29 runs over all pairs ⟨*m*,*n*⟩ of nearest neighbors, as well as for the isotropic *J*_1_–*J*_2_ model, eq 30

30 $$H=J_{1}\sum_{\left\langle m,n\right\rangle }s_{m}\cdot s_{n}+J_{2}\sum_{\left\langle \left\langle m,n\right\rangle \right\rangle }s_{m}\cdot s_{n}$$

which includes both antiferromagnetic NN (*J*_1_ > 0) and next-nearest-neighbor (NNN) couplings (*J*_2_ > 0). We cover local spins of up to *s* = 3 in selected cases. Rings, chains, and square lattices with open (OBC) or periodic (PBC) boundary conditions are chosen so that their ground-state energies are available from (sparse-matrix) ED techniques or Bethe-ansatz calculations, or from results reported in the literature. (Heisenberg chains or rings are integrable via the Bethe ansatz for $s=\frac{1}{2}$ , though not for $s>\frac{1}{2}$ , and neither are two-dimensional lattices Bethe-ansatz integrable, independent of *s*.) In addition, for rings and an icosidodecahedron, we compare field-dependent magnetization curves (which are determined by energy differences between different Fermion-number sectors) against exact results.

### 3.1 Isotropic Rings

To demonstrate that size-consistent variational oo-uLAST outperforms correlated variational approaches that lack size consistency for large enough systems, we specifically compare to earlier PHF results for Heisenberg spin rings.^8^ Table 1 compares oo-uLAST energies with reference values from ED or density-matrix renormalization group (DMRG) calculations (taken from ref 38 or ref 39) for the *S* = 0 ground states of antiferromagnetic (*J* = 1) rings with on-site spins $s=\frac{1}{2},1,\frac{3}{2},2,\frac{5}{2}$ and ring sizes *N* = 6, 12, 18, 24, 30. The largest system, *N* = 30, $s=\frac{5}{2}$ , comprises 150 auxiliary spin-1/2 particles, significantly exceeding the ∼16 such particles that could previously be treated within a full configuration-interaction (CI) basis.^25^

**Table 1:** Ground-State Energies of Antiferromagnetic Heisenberg Rings from oo-uLAST and ED (or DMRG)^a^

	*N*					
*s*	6	12	18	24	30	method
1/2	–2.803	–5.387	–8.023	–10.670	–13.322	exact
	–2.667	–5.193	–7.782	–10.375	–12.969	oo-uLAST
1	–8.617	–16.870	–25.242	–33.641	–42.046	exact/DMRG
	–7.959	–15.886	–23.904	–31.953	–39.993	oo-uLAST
3/2	–17.393	–34.131	–51.031	–67.968	–84.919	exact/DMRG
	–16.163	–32.352	–48.651	–64.976	–81.335	oo-uLAST
2	–29.165	–57.408	–85.873	–114.390	–142.927	exact/DMRG
	–27.387	–54.817	–82.383	–109.979	–137.586	oo-uLAST
5/2	–43.935	–86.679	–129.703	–172.793	–215.909	exact/DMRG
	–41.612	–83.286	–125.117	–166.962	–208.667	oo-uLAST

a For systems that were too large for ED, DMRG energies were taken from ref 39. All ED/DMRG entries agree with Table 5.1 in the latter work.

Figure 1 compares the fractional ground-state energy, *p* = *E*_method_/*E*_exact_, for oo-uLAST to PHF (employing spin and point-group symmetry, data taken from ref 8). For each *s*, the oo-uLAST curves increase smoothly with *N* and appear to converge around *p* = 0.97–0.99, although definitive conclusions would require the consideration of larger rings. These observations highlight the inherent size extensivity of oo-uLAST. By contrast, the PHF results are exact or nearly exact for *N* = 6 for all *s* values but decline monotonically as *N* increases, manifesting the lack of size extensivity.^7,40^ The energy gained from symmetry restoration scales sublinearly with system size, resulting in a progressive dilution of the recovered correlation energy as additional sites are introduced. This effect is most pronounced for $s=\frac{1}{2}$ (compare *N* = 6 to *N* = 30) but remains evident in the more classical regime of larger *s* values; for $s=\frac{5}{2}$ , oo-uLAST surpasses PHF around *N* = 24.

**Figure 1:**
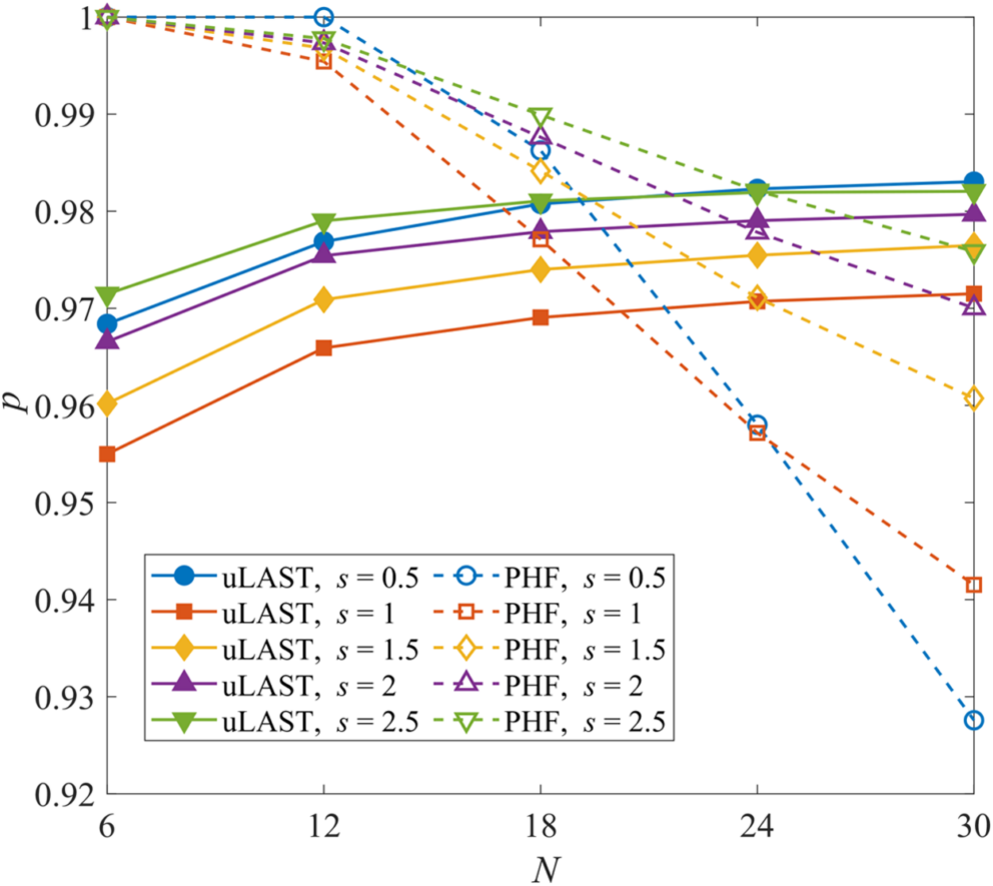
Fractional ground-state energy from oo-uLAST or PHF for the Heisenberg rings. See text for details.

### 3.2 *XXZ* Model

We consider several one- and two-dimensional systems with different on-site spins *s*. To obtain the optimal oo-uLAST solution across the range −2 ≤ Δ ≤ 2, we first independently converge solutions, using random initial guesses, for selected Δ values. From each of these points, we proceed stepwise in both directions, always using the solution from the neighboring Δ value as an initial guess. For each Δ, the solution with the lowest energy serves as the reference state for a similarity-transformed LAST calculation (the same procedure is applied for the parameter scans in the *J*_1_–*J*_2_ model; see the following section).

The JW representation reduces the strong correlation characteristic of the spin domain so that even a single Slater determinant may serve as a reasonable approximation to the ground state. This was demonstrated in ref 17, where the overlap between the exact ground state and the JW-HF wave function was computed in *XXZ* chains. In Figure 2, we additionally examine the respective overlap for oo-uLAST solutions in *N* = 6 and *N* = 12 spin-1/2 chains. The maximum configuration-interaction (CI) coefficient is defined as the absolute value of the overlap between a mean-field wave function (JW-HF or oo-uLAST) and the exact ground state. In the spin representation, this coefficient is taken to represent the largest possible overlap between any collinear spin configuration and the ground state. We do not illustrate the dependence of the JW-HF energy on the numbering scheme for spin-1/2 sites,^25^ and instead compare oo-uLAST only to JW-HF based on a sequential numbering along the chain. A maximal CI coefficient close to 1 indicates that a single determinant represents a qualitative reasonable approximation. For Δ < 0, oo-uLAST is distinct from JW-HF (thus yielding better energies), and its overlap with the exact solution is larger. In the limits of large positive or negative Δ, the ground state becomes 2-fold degenerate (the largest CI coefficient therefore approaches 1/√2), which we resolve in the ED routine by adapting the Hamiltonian to spin-flip symmetry.

**Figure 2:**
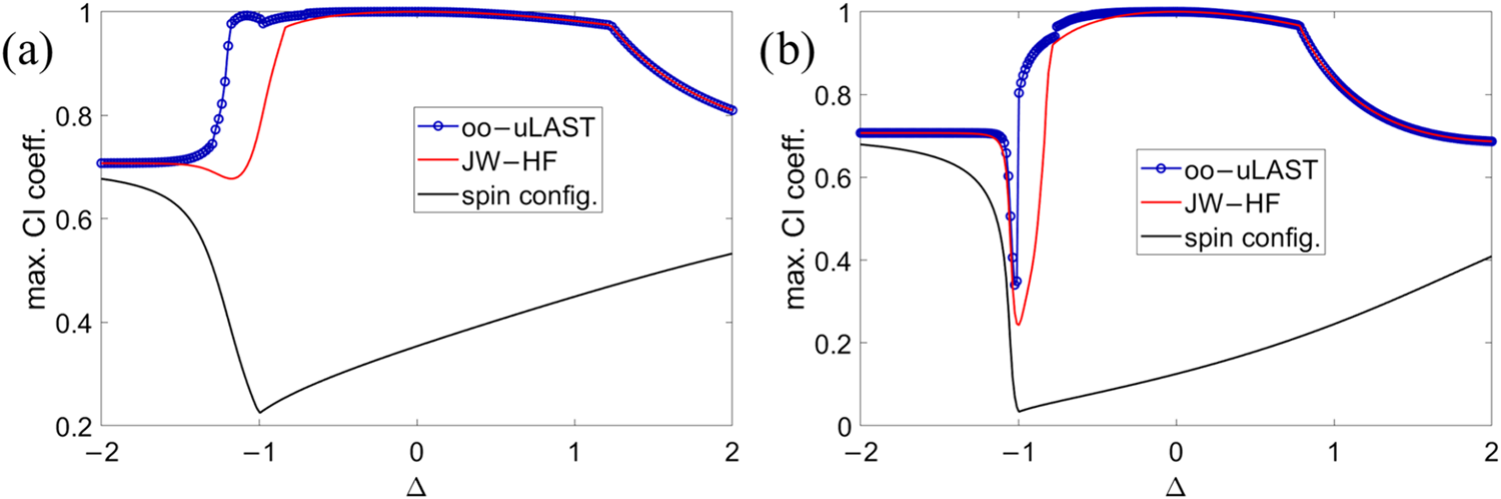
Maximum CI coefficients as functions of Δ for *XXZ* chains with *N* = 6 (a) or *N* = 12 (b) spin-1/2 sites. See text for details.

Progressions of local spin values, $s=\frac{1}{2},1,\frac{3}{2},2,\frac{5}{2},3$ , are studied for a chain and a ring with *N* = 4 centers in Figures 3 and 4, respectively, which show relative energy errors, (*E*_method_ – *E*_exact_)/*E*_exact_, in the *M* = 0 sector as a function of Δ. Note that, from a physical perspective, the *XXZ* model is not necessarily the most appropriate or complete anisotropic spin model for *s* ≥ 1 systems. In such cases, axial zero-field splitting terms, *D_m_* (*s*_*m*_^*z*^)^2^, could be incorporated straightforwardly into the mean-field framework but are omitted here for simplicity.

**Figure 3:**
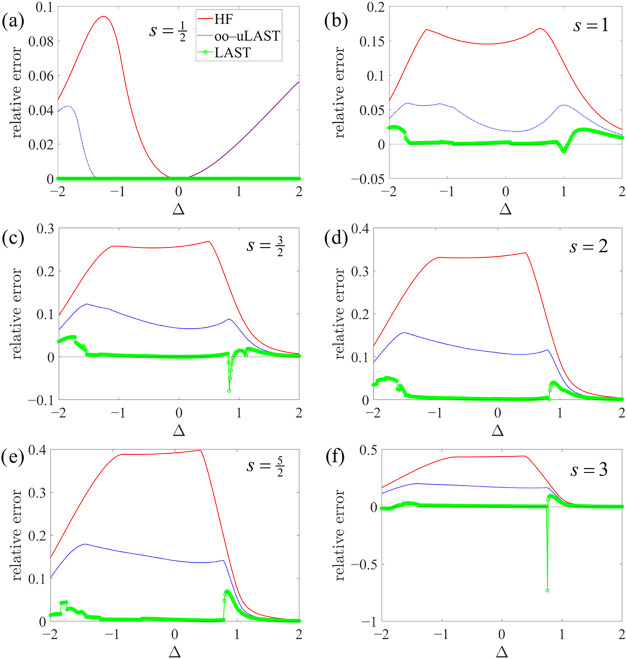
Relative energy errors for an *N* = 4 chain as a function of Δ in the *XXZ* model for different on-site spin values *s*.

**Figure 4:**
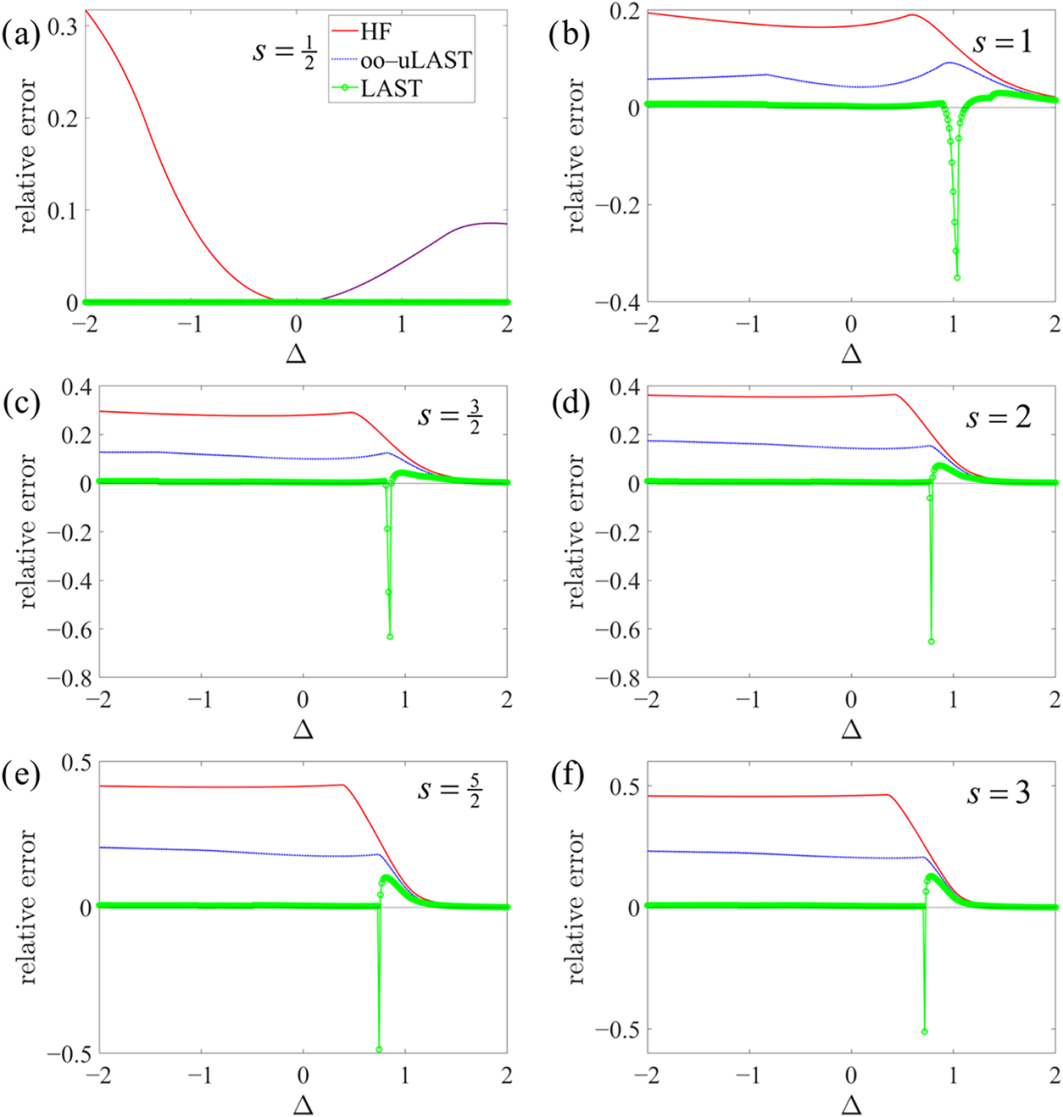
Relative energy errors for an *N* = 4 ring as a function of Δ in the *XXZ* model with different on-site spin values *s*.

We also illustrate the JW-HF energies in Figures 3 and 4. As shown in Figure 5, we first number the auxiliary spin-1/2 particles within a given center before proceeding to the next center. In general, however, this numbering (which is inconsequential for oo-uLAST) of the (auxiliary) spin-1/2 particles cannot be expected to be optimal for HF calculations within the standard JW transformation. In fact, we observe an equivalence between oo-uLAST and JW-HF only for the spin-1/2 ring and chain for Δ ≥ 0. Strictly speaking, this does not rule out the possibility that, in other systems or for different values of Δ, oo-uLAST may still become equivalent to JW-HF with an alternative numbering of the spin-1/2 particles, a point we have not investigated here. For *N* sites, there are (2*sN*)! possible orderings of the auxiliaries. Despite intrasite permutations and permutations from lattice symmetries (mirror reflection in open chains, dihedral group *D*_*N*_ in rings, etc.) being inconsequential, the number of inequivalent permutations is too large to examine individually in all but the smallest systems.

**Figure 5:**
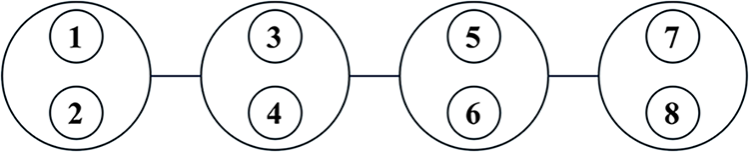
As a basis for a standard JW transformation, we assign consecutive numbers to the auxiliary spins associated with $s=\frac{1}{2}$ sites (illustrated here for a chain with four *s* = 1 sites).

In many instances, the LAST energy curves as a function of Δ exhibit numerous discontinuities and kinks, whereas JW-HF and oo-uLAST show virtually no discontinuities and significantly fewer kinks (the latter more so for oo-uLAST than for JW-HF) at points where different mean-field solutions cross, an observation consistent with earlier findings.^25^ Since the resolution of the parameter scans is necessarily limited, we cannot make a definite statement as to whether jumps in the LAST energy curves correspond to genuine discontinuities or merely to sharp transitions; such irregularities should originate from branch switches between nearly degenerate oo-uLAST references and from local convergence of the nonvariational LAST equations. Cleaner scans may be obtainable by continuation seeding of the amplitudes and tracking a fixed reference, optionally combined with step damping, but we did not pursue these refinements here. Instead, our present protocol takes the energetically lowest oo-uLAST solution for a given Δ as the reference, and LAST amplitudes **α** are initialized at zero. Thus, convergence to a local solution rather than a global solution cannot be excluded. Starting from different **α** guesses or taking an alternative, energetically nearby oo-uLAST reference could lead to a better approximation to the energy and may yield a smoother curve. Indeed, it had already been noted in ref 25 that several oo-uLAST solutions often coexist, and there is no guarantee that the lowest of them provides the optimal reference for LAST. A more exhaustive strategy of optimizing all parameters simultaneously (the uLAST and LAST parameters, **θ** and **α**, respectively, and the Slater determinant |Φ⟩) is, however, beyond the scope of the present work.

In any case, LAST typically recovers a substantial fraction of the remaining correlation energy. However, being nonvariational, it occasionally collapses to spurious solutions below the exact ground state for *s* ≥ 1 systems near the isotropic point Δ = 1, most notably for the ring in Figure 4. The problematic region, however, becomes progressively narrower with increasing *s*: from *s* = 1 to *s* = 3, its extent shrinks such that, at our scan resolution, only a single point is affected for $s=\frac{5}{2}$ and *s* = 3. Apart from this issue, LAST is virtually exact across the entire range for $s=\frac{1}{2}$ , and for larger *s* it remains highly accurate over a broad window around Δ = 0, where, particularly for higher spin values, the region following the irregularity near Δ = 1 is again described accurately at larger Δ. Note that for $s>\frac{1}{2}$ , the JW (or EJW) mapping introduces nonvanishing string operators even for a chain. As a result, at Δ = 0, the *XXZ* Hamiltonian no longer reduces to an effective noninteracting problem and thus admits no exact mean-field solution, in contrast to $s=\frac{1}{2}$ chains.

Figure 6 presents results for systems with 24 $s=\frac{1}{2}$ sites: a chain, a ring, and increasingly compact square lattices, namely 12 × 2, 8 × 3, and 6 × 4, each studied with both OBC and PBC. The JW-HF results in *n*_*x*_ × *n*_*y*_ systems are based on a numbering according to eq 31, same as eq 37 in ref 25

31 $$i_{x,y}=x+\left(y-1\right)n_{x}$$

where *n*_*x*_ > *n*_*y*_. The chain and the ring are noninteracting at Δ = 0, such that JW-HF with sequential site numbering (which eliminates the strings) is exact at this point, as are oo-uLAST and LAST. In addition, oo-uLAST appears to be exact also for the 12 × 2 systems at Δ = 0. Overall, two-dimensional lattices are described less accurately than one-dimensional systems, and relative errors tend to be larger for PBC than for OBC. For instance, the relative error of oo-uLAST remains below 3% for the chain, whereas it exceeds 10% for the 6 × 4 PBC lattice near Δ = −1. The large errors near Δ = −1 reflect an instability in the HF wave function toward the number-symmetry-broken Hartree–Fock–Bogoliubov (HFB),^16^ and in fact JW-HFB is exact at Δ = −1. We note that number-symmetry breaking in the Fermionic representation is equivalent to *S*_*z*_ symmetry breaking in the spin representation, and the difficulties at Δ = −1 presumably reflect the fact that different *S*_*z*_ sectors are degenerate at this point. We can eliminate this unphysical symmetry breaking by number projection, but since this projection is not extensive, we have limited ourselves to JW-HF in this work.

**Figure 6:**
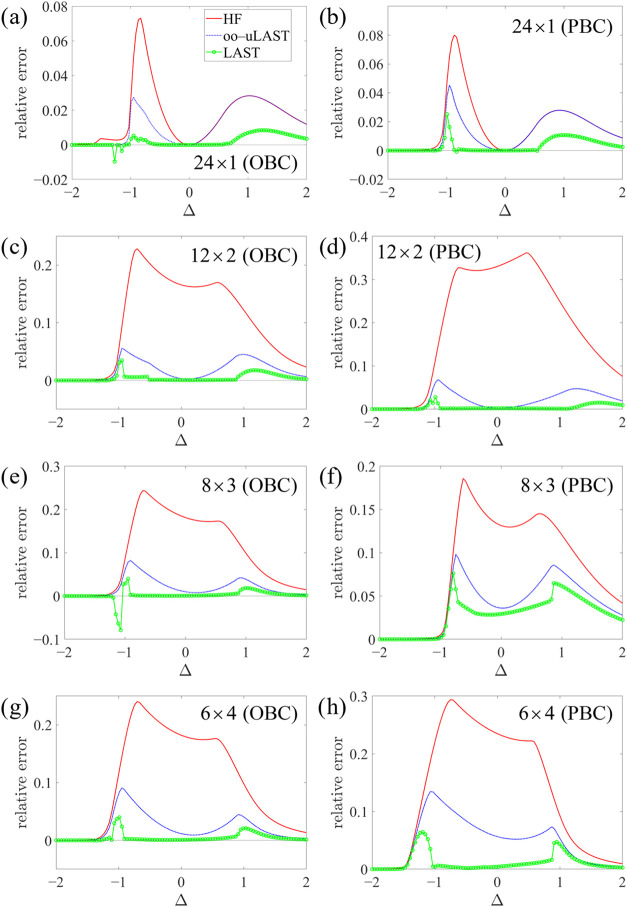
Relative energy errors for the *XXZ* model with 24 $s=\frac{1}{2}$ sites as a function of Δ.

### 3.3 *J*_1_–*J*_2_ Model

For the same lattices with 24 $s=\frac{1}{2}$ sites that were used for the *XXZ* model in the previous section, results for the relative energy errors in the *J*_1_–*J*_2_ model are plotted in Figure 7. For the chain, the LAST curve is notably jittery, reflecting multiple competing solutions; we have attempted to follow the lowest-energy branch by seeding each point with the converged solution at the neighboring value of *J*_2_/*J*_1_, as described above for oo-uLAST. Despite this piecewise behavior, LAST is very accurate in a neighborhood of the Majumdar–Ghosh point *J*_2_/*J*_1_ = 0.5,^41^ where JW-HF is exact (in the chain and the ring) due to dimer formation. A striking positive case is the 12 × 2 PBC lattice, where LAST is essentially exact over a wide interval, extending up to *J*_2_/*J*_1_ ≈ 0.85. By contrast, on other lattices, LAST exhibits more irregular features (cusps and small discontinuities), with a pronounced dip for 6 × 4 PBC around *J*_2_/*J*_1_ ≈ 0.65 that crosses slightly below zero error (nonvariational behavior).

**Figure 7:**
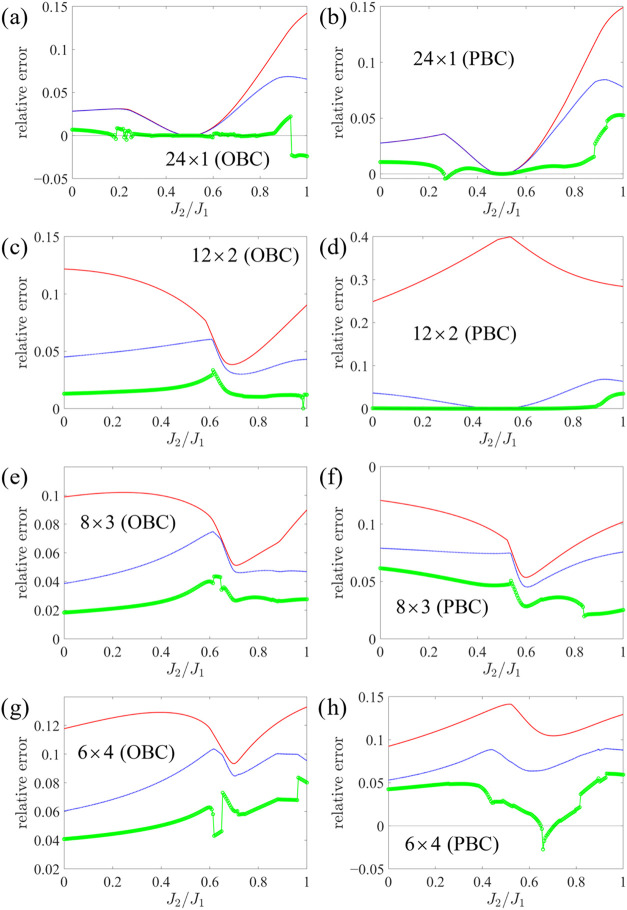
Relative energy errors for systems with 24 $s=\frac{1}{2}$ sites as a function of *J*_2_/*J*_1_ in the antiferromagnetic (*J*_1_ = 1) *J*_1_–*J*_2_ model. The lattices are the same as those in Figure 6.

Overall, oo-uLAST provides far smoother curves, consistently better than JW-HF (again, except for the chain and the ring, where oo-uLAST is equivalent to JW-HF for *J*_2_/*J*_1_ ≤ 0.5), and less erratic than LAST. As observed on the respective lattices in the *XXZ* model, two-dimensional lattices or PBC typically yield larger errors, with the 12 × 2 PBC exception highlighted above.

Finally, results for 8 × 2 and 4 × 4 lattices composed of *s* = 1 centers are shown in Figure 8, both with the OBC or PBC. For 8 × 2 with the OBC, all three curves run approximately in parallel. In general, however, the LAST curves display markedly larger irregularities than for comparable spin-1/2 systems, which is especially true for the 4 × 4 OBC case. The best description is obtained for 8 × 2 with PBC, where within an interval around *J*_2_/*J*_1_ ≈ 0.45, LAST is nearly exact.

**Figure 8:**
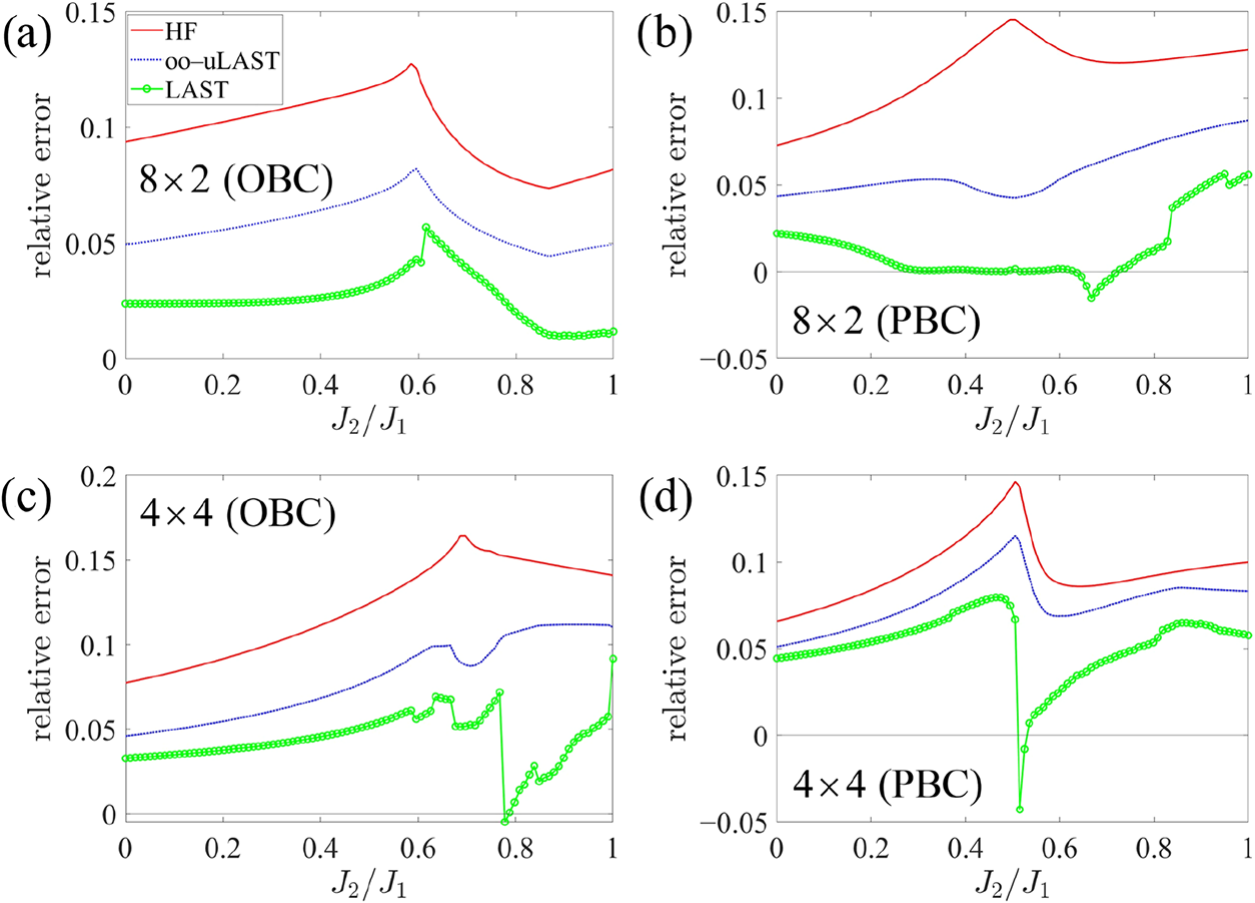
Relative energy errors for systems with 16 *s* = 1 sites in the antiferromagnetic *J*_1_–*J*_2_ model.

### 3.4 Field-Dependent Magnetization

So far, we have worked within the *M* = 0 sector, which corresponds to half-filling in the JW representation. Here, we consider the field-dependent magnetization for antiferromagnetic isotropic $s=\frac{1}{2}$ rings and the icosidodecahedron, which is determined by energy differences between *M* sectors (Fermion-number sectors). At zero temperature, the thermal average is ⟨M⟩=gM , where *g* is the gyromagnetic factor and *M* is the *S*_*z*_ eigenvalue of the field-dependent global ground state. The levels *E*_*M*_ = *E*_*M*_^(0)^–*g*μ_*B*_ *BM* comprise a field-independent term *E*_*M*_^(0)^ and a Zeeman contribution (we set *g* = 2 and μ_*B*_ = 1). The magnetization curve is obtained from ground-state energies *E*_*M*_^(0)^ in the fixed-*M* sectors. A step *M* → *M* + 1 occurs at *B* = (*E*_*M*+1_^(0)^ – *E*_*M*_^(0)^)/(*g*μ_*B*_). Hence, the staircase directly reflects differences in sector energies. Magnetization curves are routinely measured for exchange-coupled spin clusters and can be used to fit model parameters (e.g., coupling constants). Figures 9 and 10 display ⟨M⟩ as a function of the magnetic field *B* for *N* = 30 and *N* = 60 rings, respectively. The exact curves were obtained from Bethe-ansatz energies computed using the ABACUS^42^ program. In both systems, oo-uLAST reproduces the staircase structure only qualitatively: the first step from *M* = 0 to *M* = 1, corresponding to the singlet–triplet gap, is shifted to a significantly higher field, which compresses the spacing between the first and second step (at *B* ≈ 0.15 for *N* = 60). Adding correlation via LAST removes this defect almost completely for *N* = 30, and LAST predictions for *N* = 60 are also rather accurate.

**Figure 9:**
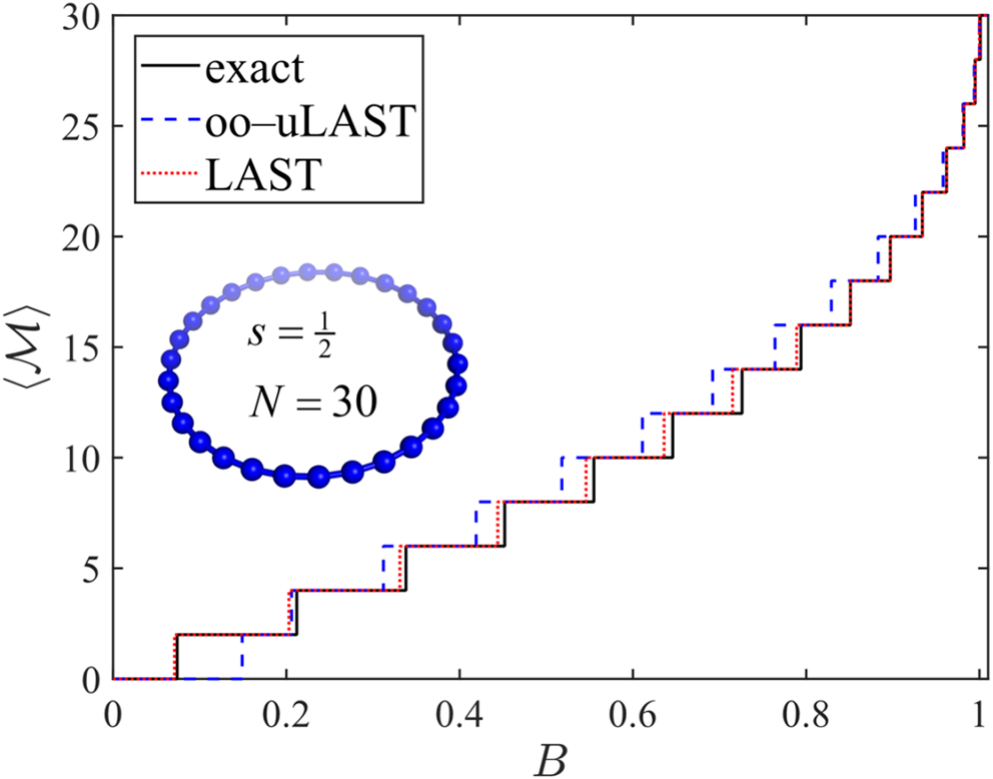
Field-dependent zero-temperature magnetization for an $s=\frac{1}{2}$ ring with *N* = 30 sites in the antiferromagnetic Heisenberg model (spheres and connecting lines represent spins and pairwise couplings, respectively).

**Figure 10:**
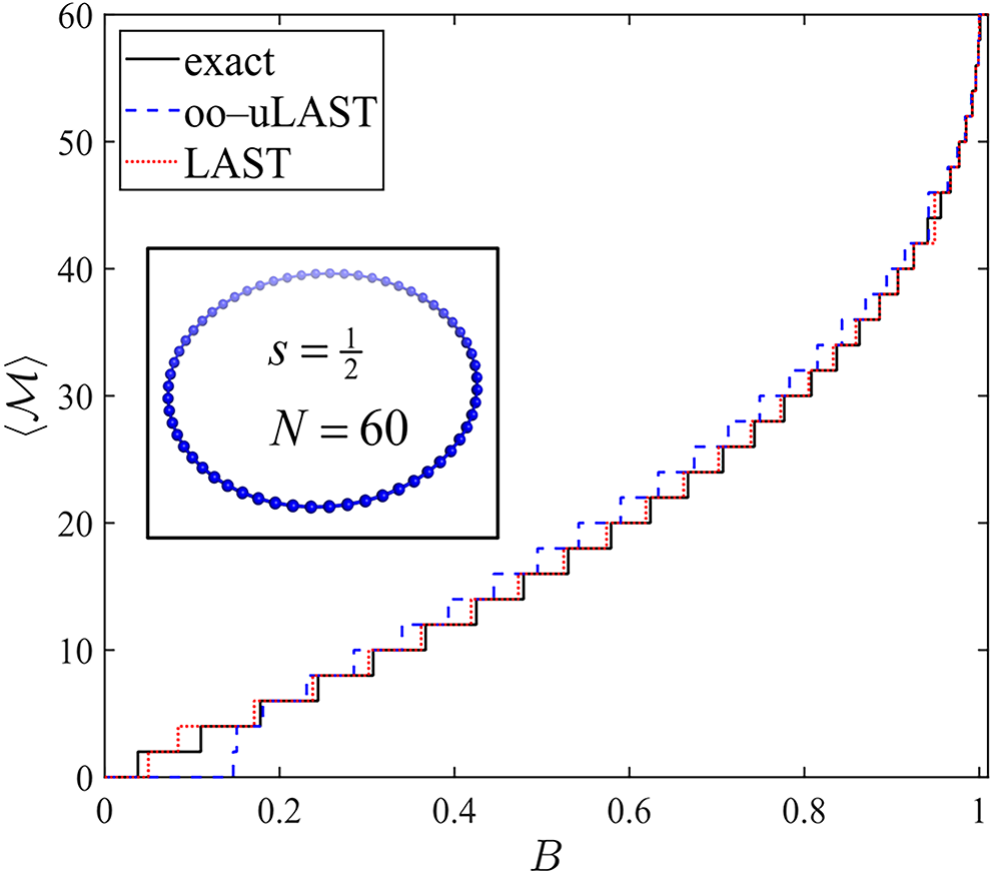
Field-dependent zero-temperature magnetization for an antiferromagnetic $s=\frac{1}{2}$ ring with *N* = 60 sites.

Finally, Figure 11 shows the magnetization staircase for an antiferromagnetic icosidodecahedron. The exact curve was calculated from the ground-state energies in all *M* sectors reported by Rousochatzakis et al., cf. Table Ib and Figure 5 in ref 43. Errors of oo-uLAST as well as LAST are significantly larger than those in rings. The most prominent feature is the plateau at ⟨M⟩=10 (one-third of the saturation value). Its width is significantly overestimated by oo-uLAST but captured more accurately by LAST. Away from the plateau region, LAST generally continues to track the exact staircase more closely.

**Figure 11:**
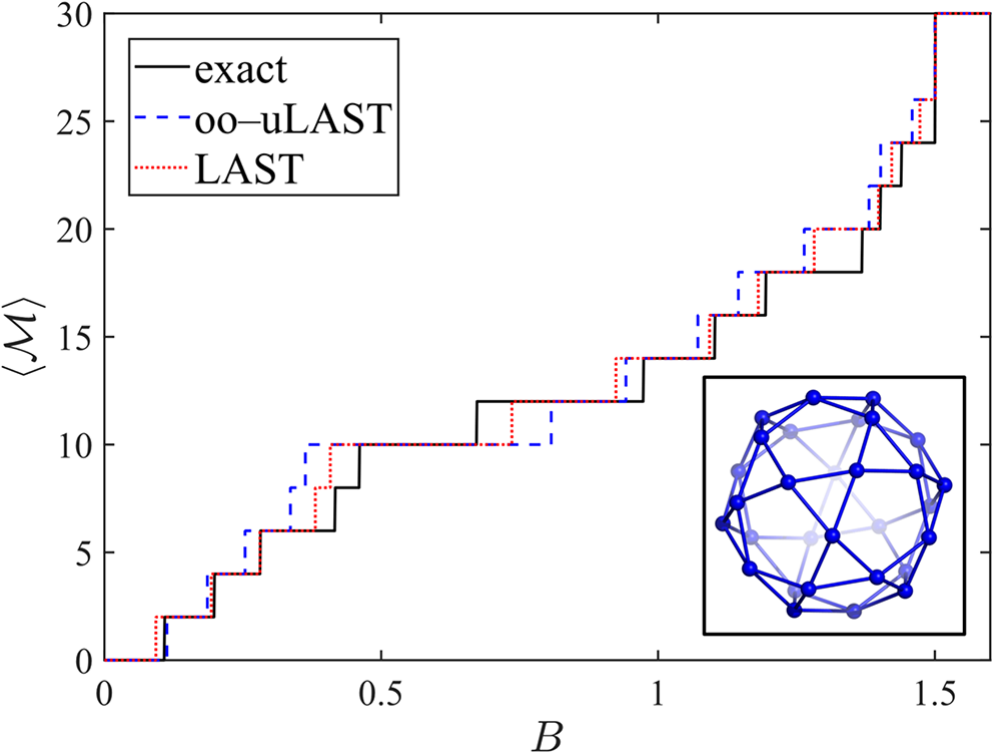
Zero-temperature magnetization as a function of magnetic field for the $s=\frac{1}{2}$ icosidodecahedron in the antiferromagnetic Heisenberg model.

## 4 Summary and Conclusions

We have developed an efficient framework for orbital-optimized unitary Lie-Algebraic Similarity Transformation (oo-uLAST) and its size-extensive nonunitary extension (LAST) for spin Hamiltonians, based on mapping spins to Fermions via an extended Jordan–Wigner (EJW) transformation. The analytical energy gradients and the Jacobian derived here make the combined optimization of the mean-field state and EJW angles, and the subsequent determination of LAST amplitudes, computationally feasible, even for systems with >100 spin-1/2 particles. We suggested an auxiliary-qubit decomposition that makes $s>\frac{1}{2}$ systems amenable to Fermionization by EJW. This constitutes a particularly simple scheme, requiring only very minor changes relative to an $s=\frac{1}{2}$ implementation. Although the mapping enlarges the Hilbert space, the procedure is variationally safe: as we prove here, the ground state of a bilinear spin Hamiltonian carries maximal local spins.

Our results confirm that the spin-Fermion mapping weakens strong correlation compared to that of the spin domain. In particular, the variational and size-extensive oo-uLAST approach removes the ambiguity of site ordering and recovers a significant fraction (≈97–99%) of the exact ground-state energy for Heisenberg rings up to (and beyond) *N* = 30 sites and for various local spin values (at present up to $s=\frac{5}{2}$ ).

In summary, the present findings further underline that the strategy of Fermionizing finite spin systems and applying simple, cost-effective many-body techniques from electronic-structure theory remains an underexplored, highly promising direction for treating strong correlation that should be pursued further. A natural extension of the present framework would be to replace the single-determinant reference with a compact linear combination of multiple determinants. For instance, an ansatz akin to resonating Hartree–Fock, involving a linear combination of a few simultaneously optimized determinants, could be straightforwardly embedded within the oo-uLAST formalism and would provide a viable starting point for correlation through similarity-transformed LAST. A direct way to construct linear combinations would be to employ symmetries. However, symmetry operations that are simple in the spin representation often map to complicated, nonlocal Fermionic operators under the JW transformation, making their implementation less straightforward. Some useful exceptions do exist, however, like the spin-flip symmetry in the *M* = 0 sector, which allows one to separate even and odd total-spin sectors, or the cyclic point-group symmetry of spin-1/2 rings. In contrast, full adaptation to total-spin eigenstates or the general exploitation of point-group symmetries in more complicated lattices may not always be practical within the Fermionic framework.

## Appendix

### A1 Optimization of the Mean-Field State

Each column of the matrix **C** defines a molecular orbital (MO), eq 32

A1
$$a_{i}^{†}=\sum_{m}C_{\mathrm{mi}}c_{m}^{†}$$

There are (*N*_orb_ – *N*_*f*_) × *N*_*f*_ complex parameters *Z*_*vo*_, where *N*_orb_ is the number of single-particle basis functions (the number of spin-1/2 particles) and *N*_*f*_ is the number of Fermions. The transformation of an initial guess **C** ^0^ into **C** proceeds through the intermediate Ċ [compare eqs 33 and 34 to eqs 3.45 and 3.47 in ref 44]­

A2
$$\dot{C_{o}\;}=C_{o}^{0}+\sum_{v}Z_{\mathrm{vo}}C_{v}^{0}$$

A3
$$\dot{C_{v}\;}=C_{v}^{0}-\sum_{o}Z_{\mathrm{vo}}^{*}C_{o}$$

where $\dot{C_{o}\;}$ is the *o*th column of Ċ , etc. Equations 35 and 36 form the orthonormal **C**

A4
$${\left(C_{\mathrm{occ}}\right)}_{\mathrm{lm}}=\sum_{k}{\left(L^{-1}\right)}_{\mathrm{lk}}{\left(\dot{C_{\mathrm{occ}}\;}\right)}_{\mathrm{km}}$$

A5
$${\left(C_{\mathrm{virt}}\right)}_{\mathrm{lm}}=\sum_{k}{\left(K^{-1}\right)}_{\mathrm{lk}}{\left(\dot{C_{\mathrm{virt}}\;}\right)}_{\mathrm{km}}$$

using lower-triangular matrices **L** and **K** from the Cholesky decompositions of eqs 37 and 38

A6
$$1+Z^{T}Z^{*}=LL^{†}$$

A7
$$1+Z^{*}Z^{T}=KK^{†}$$

Energy and gradient evaluations require single- and two-particle matrix elements between the nonorthogonal determinants |Φ⟩ and |Φ̃⟩. The respective expressions utilize the single-particle transition-density matrix **ρ** ^ΦΦ̃^ with elements ρ_*kl*_^ΦΦ̃^

$$\rho_{\mathrm{kl}}^{\Phi \mathrm{\Phi ̃}}\equiv \frac{\langle \Phi |c_{l}^{†}c_{k}|\mathrm{\Phi ̃}\rangle }{\langle \Phi \left|\mathrm{\Phi ̃}\right\rangle }={\left({\mathrm{C̃}}_{\mathrm{occ}}M^{-1}C_{\mathrm{occ}}^{†}\right)}_{\mathrm{kl}}$$
A8

where **M** ≡ **C**_occ_^†^ **C̃**_occ_; the overlap is ⟨Φ|Φ̃⟩ = det­(**M**). For a derivation of eq 39, see, e.g., ref 44. The two-particle matrix elements are an antisymmetrized product, eq 40; to avoid clutter, we use ρ_*mk*_ instead of ρ_*mk*_^ΦΦ̃^

A9
$$\frac{\langle \Phi |c_{k}^{†}c_{l}^{†}c_{m}c_{n}|\mathrm{\Phi ̃}\rangle }{\langle \Phi \left|\mathrm{\Phi ̃}\right\rangle }=\left|\begin{array}{ll} \rho_{\mathrm{mk}} & \rho_{\mathrm{ml}}\\ \rho_{\mathrm{nk}} & \rho_{\mathrm{nl}} \end{array}\right|=\rho_{\mathrm{mk}}\rho_{\mathrm{nl}}-\rho_{\mathrm{ml}}\rho_{\mathrm{nk}}$$

For |Φ̃⟩ = |Φ⟩, the single-particle density matrix is **ρ** = **C**_occ_ **C**_occ_^†^ and the evaluation of the *z*-coupling energy is standard, see eq 41

A10
$$E_{z}\equiv \left\langle \Phi \right|H_{z}\left|\Phi \right\rangle =E_{\mathrm{const}}+\mathrm{tr(}t\rho )+\frac{1}{2}\mathrm{tr(}\Gamma \rho )$$

with **Γ** defined in eq 42

A11
$$\Gamma_{\mathrm{kl}}=\sum_{\mathrm{mn}}\left[\mathrm{kl}|\mathrm{mn}\right]\rho_{\mathrm{nm}}$$

The *x*- and *y*-coupling contributions are slightly more complicated due to the presence of strings, which act as Thouless rotations.^17^ As an example, for |Φ̃⟩ = *e* ^*i*Σ_*q*_θ_*pq*_ *n*_*q*_^|Φ⟩, it is straightforward to verify eq 43

A12
$$\mathrm{C̃}=\left(\begin{array}{lll} e^{i\theta_{p1}} & 0 & 0\\ 0 & \ddots & 0\\ 0 & 0 & e^{i\theta_{\mathrm{pN}}} \end{array}\right)C$$

This is indeed a special case of eq 6 in ref 26. For each interacting pair ⟨*m*,*n*⟩, we thus consider |Φ̃_*mn*_⟩ = ϕ_*m*(*n*)_^†^ϕ_*n*(*m*)_|Φ⟩ and evaluate the respective hopping term, $\frac{1}{2}c_{m}^{†}c_{n}$ . This overall yields the energy contribution of eq 44

A13
$$E_{\mathrm{mn}}^{\mathrm{xy}}\equiv \left\langle \Phi \right|s_{m}^{x}s_{n}^{x}+s_{m}^{y}s_{n}^{y}\left|\Phi \right\rangle =\frac{1}{2}\mathrm{tr}\left(h_{\mathrm{mn}}\rho^{\Phi {\mathrm{\Phi ̃}}_{\mathrm{mn}}}\right)\left\langle \Phi \right|{\mathrm{\Phi ̃}}_{\mathrm{mn}}\rangle +c\mathrm{.c}.$$

where (**h** *_mn_*)_*ij*_ = *J*_*mn*_δ_*mi*_δ_*nj*_.

In the context of PHF for electronic-structure theory, a detailed derivation of the gradient with respect to the Thouless parameters can be found in ref 44, which also cites earlier work on gradient-based mean-field calculations. The global gradient G is defined in terms of energy variations with respect to Thouless rotations applied to the reference |Φ^0^⟩. Its elements $G_{\mathrm{vo}}$ are defined through eq 45, where the real and imaginary parts of *Z*_*vo*_ are independent variables.

A14
δE=∑vo∂E∂Zvo*δZvo*+c.c.=−∑vo(GvoδZvo*+c.c.)=−2∑vo[Re(Gvo)Re(δZvo)+Im(Gvo)Im(δZvo)]

We explicitly calculate the local gradient **G** and ultimately transform it into the MO-basis of the initial guess |Φ^0^⟩ to obtain the global gradient, see eq 46

A15
$$G={\left(C_{\mathrm{virt}}^{0}\right)}^{†}GC_{\mathrm{occ}}^{0}$$

The respective contributions of the decomposition in eq 47

A16
$$G=G_{z}+\sum_{m<n}G_{\mathrm{mn}}^{\mathrm{xy}}$$

are given in eqs 48 and 49.

A17
$${\left(G_{z}\right)}_{\mathrm{vo}}=-\left[\left(\rho -1\right)\left(t+\Gamma \right)-\left(E_{z}-E_{\mathrm{const}}\right)\right]\rho$$

A18
$$G_{\mathrm{mn}}^{\mathrm{xy}}=-\frac{1}{2}\left[\left(\rho^{\Phi {\mathrm{\Phi ̃}}_{\mathrm{mn}}}-1\right)h_{\mathrm{mn}}-\mathrm{tr(}h_{\mathrm{mn}}\rho^{\Phi {\mathrm{\Phi ̃}}_{\mathrm{mn}}})\right]\rho^{\Phi {\mathrm{\Phi ̃}}_{\mathrm{mn}}}\left\langle \Phi \right|{\mathrm{\Phi ̃}}_{\mathrm{mn}}\rangle +h.c.$$

### A2 uLAST Gradient

Here, to optimize the uLAST parameters θ_*pq*_ efficiently, we derive the gradient ϑ defined in eq 50

A19
$$\vartheta_{\mathrm{pq}}\equiv \frac{\partial \left\langle \Phi \right|H\left(\theta \right)\left|\Phi \right\rangle }{\partial \theta_{\mathrm{pq}}}=\left\langle \Phi \right|\frac{\partial H\left(\theta \right)}{\partial \theta_{\mathrm{pq}}}\left|\Phi \right\rangle$$

The derivative of the combined *x*- and *y*-coupling terms for a pair ⟨*m*,*n*⟩ is considered in eq 51.

A20
$$\frac{\partial }{\partial \theta_{\mathrm{pq}}}\left(s_{m}^{x}s_{n}^{x}+s_{m}^{y}s_{n}^{y}\right)=\frac{\partial }{\partial \theta_{\mathrm{pq}}}\left[\frac{1}{2}c_{m}^{†}c_{n}\phi_{m\left(n\right)}^{†}\phi_{n\left(m\right)}+h.c.\right]=\frac{1}{2}c_{m}^{†}c_{n}\left[\phi_{n\left(m\right)}\frac{\partial \phi_{m\left(n\right)}^{†}}{\partial \theta_{\mathrm{pq}}}+\phi_{m\left(n\right)}^{†}\frac{\partial \phi_{n\left(m\right)}}{\partial \theta_{\mathrm{pq}}}\right]+h.c.$$

For one term on the right-hand side of eq 51, the calculation is exemplarily detailed in eq 52, where θ_*qp*_ = θ_*pq*_ + π for *p* < *q*.

A21
$$\frac{\partial \phi_{n\left(m\right)}}{\partial \theta_{\mathrm{pq}}}=\frac{\partial e^{-i\sum_{r\neq m}\theta_{\mathrm{nr}}n_{r}}}{\partial \theta_{\mathrm{pq}}}=-i\frac{\partial \left[\theta_{\mathrm{nq}}n_{q}\left(1-\delta_{\mathrm{mq}}\right)+\theta_{\mathrm{np}}n_{p}\left(1-\delta_{\mathrm{mp}}\right)\right]}{\partial \theta_{\mathrm{pq}}}\phi_{n\left(m\right)}=-i\frac{\partial \left[\theta_{\mathrm{nq}}n_{q}\left(1-\delta_{\mathrm{mq}}\right)+\left(\theta_{\mathrm{pn}}\pm \pi \right)n_{p}\left(1-\delta_{\mathrm{mp}}\right)\right]}{\partial \theta_{\mathrm{pq}}}\phi_{n\left(m\right)}=-i\left[\delta_{\mathrm{np}}\left(1-\delta_{\mathrm{mq}}\right)n_{q}+\delta_{\mathrm{nq}}\left(1-\delta_{\mathrm{mp}}\right)n_{p}\right]\phi_{n\left(m\right)}$$

A similar calculation yields eq 53

A22
$$\frac{\partial \phi_{m\left(n\right)}^{†}}{\partial \theta_{\mathrm{pq}}}=i\left[\delta_{\mathrm{mp}}\left(1-\delta_{\mathrm{nq}}\right)n_{q}+\delta_{\mathrm{mq}}\left(1-\delta_{\mathrm{np}}\right)n_{p}\right]\phi_{m\left(n\right)}$$

and we overall obtain eq 54.

A23
$$\frac{\partial }{\partial \theta_{\mathrm{pq}}}\left[\frac{1}{2}c_{m}^{†}c_{n}\phi_{m\left(n\right)}^{†}\phi_{n\left(m\right)}+h.c.\right]=\frac{i}{2}\left\{c_{m}^{†}c_{n}\left[\delta_{\mathrm{mp}}\left(1-\delta_{\mathrm{nq}}\right)-\delta_{\mathrm{np}}\left(1-\delta_{\mathrm{mq}}\right)\right]n_{q}+\left[\delta_{\mathrm{mq}}\left(1-\delta_{\mathrm{np}}\right)-\delta_{\mathrm{nq}}\left(1-\delta_{\mathrm{mp}}\right)\right]n_{p}\right\}\phi_{m\left(n\right)}^{†}\phi_{n\left(m\right)}+h.c.$$

Establishing normal order through eq 55

A24
$$c_{m}^{†}c_{n}n_{r}=c_{m}^{†}c_{n}c_{r}^{†}c_{r}=c_{m}^{†}\left(\delta_{\mathrm{nr}}-c_{r}^{†}c_{n}\right)c_{r}=\delta_{\mathrm{nr}}c_{m}^{†}c_{r}-c_{m}^{†}c_{r}^{†}c_{n}c_{r}$$

gives eq 56.

$$\frac{\partial }{\partial \theta_{\mathrm{pq}}}\left[\frac{1}{2}c_{m}^{†}c_{n}\phi_{m\left(n\right)}^{†}\phi_{n\left(m\right)}+h.c.\right]=\frac{i}{2}\left\{\left[\delta_{\mathrm{mp}}\left(1-\delta_{\mathrm{nq}}\right)-\delta_{\mathrm{np}}\left(1-\delta_{\mathrm{mq}}\right)\right]\left(\delta_{\mathrm{nq}}c_{m}^{†}c_{q}-c_{m}^{†}c_{q}^{†}c_{n}c_{q}\right)+\left[\delta_{\mathrm{mq}}\left(1-\delta_{\mathrm{np}}\right)-\delta_{\mathrm{nq}}\left(1-\delta_{\mathrm{mp}}\right)\right]\left(\delta_{\mathrm{np}}c_{m}^{†}c_{p}-c_{m}^{†}c_{p}^{†}c_{n}c_{p}\right)\right\}\times \phi_{m\left(n\right)}^{†}\phi_{n\left(m\right)}+h.c.$$
A25

The single-particle contributions in the curly braces of eq 56 identically vanish. Hence, only the four-Fermion terms contribute, yielding eq 57.

$$\frac{\partial \left(s_{m}\cdot s_{n}\right)}{\partial \theta_{\mathrm{pq}}}=-\frac{i}{2}\left\{\left[\delta_{\mathrm{mp}}\left(1-\delta_{\mathrm{nq}}\right)-\delta_{\mathrm{np}}\left(1-\delta_{\mathrm{mq}}\right)\right]c_{m}^{†}c_{q}^{†}c_{n}c_{q}+\left[\delta_{\mathrm{mq}}\left(1-\delta_{\mathrm{np}}\right)-\delta_{\mathrm{nq}}\left(1-\delta_{\mathrm{mp}}\right)\right]c_{m}^{†}c_{p}^{†}c_{n}c_{p}\right\}\times \phi_{m\left(n\right)}^{†}\phi_{n\left(m\right)}+h.c.$$
A26

*E*, **C** ^0^, G and ϑ are passed to fminunc, a Matlab function for unconstrained minimization. We separate terms as in eq 45, because fminunc requires real optimization parameters.

### A3 LAST Residuals and Jacobian

A string operator resulting from a general LAST (including a uLAST component) scales the orbital coefficients nonuniformly, see eq 58 for |Φ̃⟩ = *e* ^Σ_*q*_(α_*pq*_+*i*θ_*pq*_)*n*_*q*_^|Φ⟩.

A27
$${\mathrm{C̃}}_{\mathrm{occ}}=\left(\begin{array}{lll} e^{\alpha_{p1}+i\theta_{p1}} & 0 & 0\\ 0 & \ddots & 0\\ 0 & 0 & e^{\alpha_{\mathrm{pN}}+i\theta_{\mathrm{pN}}} \end{array}\right)C_{\mathrm{occ}}$$

While the columns of **C̃**_occ_ are neither normalized nor orthogonal, |Φ̃⟩ still represents a single Slater determinant up to a prefactor.^26^ Importantly, eqs 39 and 40 for the single- and two-particle matrix elements remain valid. Equation 59 shows the form of *x*- and *y*-coupling contributions to the residuals

A28
$$\left\langle \Phi \right|n_{p}n_{q}c_{m}^{†}c_{n}\phi_{m\left(n\right)}^{†}\phi_{n\left(m\right)}\left|\Phi \right\rangle$$

and eq 60 establishes normal order

A29
$$n_{p}n_{q}c_{m}^{†}c_{n}=c_{p}^{†}c_{q}^{†}c_{m}^{†}c_{q}c_{p}c_{n}-\delta_{\mathrm{mq}}c_{p}^{†}c_{q}^{†}c_{p}c_{n}+\delta_{\mathrm{mp}}c_{p}^{†}c_{q}^{†}c_{q}c_{n}$$

The three-particle term is evaluated based on the general eq 61

A30
$$\frac{\langle \Phi |c_{k}^{†}c_{l}^{†}c_{m}^{†}c_{n}c_{o}c_{p}|\mathrm{\Phi ̃}\rangle }{\langle \Phi \left|\mathrm{\Phi ̃}\right\rangle }=-\left|\begin{array}{lll} \rho_{\mathrm{nk}} & \rho_{\mathrm{nl}} & \rho_{\mathrm{nm}}\\ \rho_{\mathrm{ok}} & \rho_{\mathrm{ol}} & \rho_{\mathrm{om}}\\ \rho_{\mathrm{pk}} & \rho_{\mathrm{pl}} & \rho_{\mathrm{pm}} \end{array}\right|$$

The single-particle terms from *z*-coupling contribute terms of the form ⟨Φ|*n*_*p*_ *n*_*q*_ *n*_*m*_|Φ⟩ to the residuals, which can be evaluated similarly. For the two-particle contribution from *z*-coupling, however, we must append another number operator to the right of eq 62.

A31
$$n_{p}n_{q}n_{m}n_{n}=c_{p}^{†}c_{q}^{†}c_{m}^{†}c_{n}^{†}c_{p}c_{q}c_{m}c_{n}-\delta_{\mathrm{mn}}c_{p}^{†}c_{q}^{†}c_{m}^{†}c_{p}c_{q}c_{n}-\delta_{\mathrm{np}}c_{p}^{†}c_{q}^{†}c_{m}^{†}c_{q}c_{m}c_{n}+\delta_{\mathrm{nq}}c_{p}^{†}c_{q}^{†}c_{m}^{†}c_{p}c_{m}c_{n}-\delta_{\mathrm{mq}}\left(\delta_{\mathrm{mn}}c_{p}^{†}c_{q}^{†}c_{p}c_{n}-\delta_{\mathrm{np}}c_{p}^{†}c_{q}^{†}c_{m}c_{n}+c_{p}^{†}c_{q}^{†}c_{n}^{†}c_{p}c_{m}c_{n}\right)+\delta_{\mathrm{mp}}\left(\delta_{\mathrm{mn}}c_{p}^{†}c_{q}^{†}c_{q}c_{n}-\delta_{\mathrm{nq}}c_{p}^{†}c_{q}^{†}c_{m}c_{n}+c_{p}^{†}c_{q}^{†}c_{n}^{†}c_{q}c_{m}c_{n}\right)$$

Equation 62 contains a four-particle term, which is evaluated based on eq 63.

A32
$$\frac{\left\langle \Phi \right|c_{k}^{†}c_{l}^{†}c_{m}^{†}c_{n}^{†}c_{o}c_{p}c_{q}c_{r}\left|\mathrm{\Phi ̃}\right\rangle }{\langle \Phi \left|\mathrm{\Phi ̃}\right\rangle }=\left|\begin{array}{llll} \rho_{\mathrm{ok}} & \rho_{\mathrm{ol}} & \rho_{\mathrm{om}} & \rho_{\mathrm{on}}\\ \rho_{\mathrm{pk}} & \rho_{\mathrm{pl}} & \rho_{\mathrm{pm}} & \rho_{\mathrm{pn}}\\ \rho_{\mathrm{qk}} & \rho_{\mathrm{ql}} & \rho_{\mathrm{qm}} & \rho_{\mathrm{qn}}\\ \rho_{\mathrm{rk}} & \rho_{\mathrm{rl}} & \rho_{\mathrm{rm}} & \rho_{\mathrm{rn}} \end{array}\right|$$

The calculation of the Jacobian, eq 26 in the main text, involves the derivative of *H̅* with respect to α_*pq*_, eq 64. The steps to arrive at this result are not given here, because they are very similar to the derivation of the uLAST gradient (see previous section).

A33
$$\frac{\partial \mathrm{H̅}}{\partial \alpha_{\mathrm{pq}}}=-\frac{1}{2}\sum_{m<n}J_{\mathrm{mn}}\left\{\left[\delta_{\mathrm{mp}}\left(1-\delta_{\mathrm{nq}}\right)+\delta_{\mathrm{np}}\left(1-\delta_{\mathrm{mq}}\right)\right]c_{m}^{†}c_{q}^{†}c_{n}c_{q}+\left[\delta_{\mathrm{mq}}\left(1-\delta_{\mathrm{np}}\right)+\delta_{\mathrm{nq}}\left(1-\delta_{\mathrm{mp}}\right)\right]c_{m}^{†}c_{p}^{†}c_{n}c_{p}\right\}\phi_{m\left(n\right)}^{†}\phi_{n\left(m\right)}+h.c.$$

### A4 Proof: Auxiliary Qubits Form Maximal Local Spins in the Ground State of a Bilinear Hamiltonian

Consider a general bilinear spin Hamiltonian, eq 65, where the local spin-quantum numbers *s*_*p*_ may be different for different sites. The pairwise coupling is parametrized by 3 × 3 matrices **D**_*mn*_ (Cartesian rank-2 tensors) with arbitrary values of the real components *D*_*mn*_^αβ^

A34
$$H=\sum_{m<n}s_{m}\cdot D_{\mathrm{mn}}\cdot s_{n}=\sum_{m<n}\sum_{\alpha ,\beta =x,y,z}D_{\mathrm{mn}}^{\alpha \beta }s_{m}^{\alpha }s_{n}^{\beta }$$

In general, *H* does not conserve *S*_*z*_, which is a case not directly relevant to the mean-field calculations performed in this work. However, the *XXZ* model, which conserves *S*_*z*_, is a special case of eq 65 where **D**_*mn*_ = diag­(*J*_*mn*_, *J*_*mn*_, Δ_*mn*_) for all pairs ⟨*m*,*n*⟩.

We extend the Hilbert space as shown in eq 66

A35
$$s_{p}\to {\mathrm{s̃}}_{p}=\sum_{a=1}^{2s_{p}}\kappa_{p,a}$$

by introducing 2*s*_*p*_ spin-1/2 auxiliaries at each site. (As apparent, certain operators carry a tilde in the auxiliary-qubit space. To avoid clutter, we do not use tildes on operators that are not defined or trivial in the original space of *H*.) In the following, we prove that the ground state of *H̃*, eq 67

A36
$$H\to \mathrm{H̃}=\sum_{m<n}{\mathrm{s̃}}_{m}\cdot D_{\mathrm{mn}}\cdot {\mathrm{s̃}}_{n}$$

belongs to the space of the physical Hamiltonian *H*, i.e., all on-site spins are maximal. We are not aware of any previous proof of this proposition in the literature.

Note first that, because local spin is a good quantum number, i.e., [*H̃*, **s̃**_*p*_^2^] = 0 for each *p*, the Hilbert space splits into sectors labeled by the set of on-site spins (*t*_1_, *t*_2_, ···, *t*_*N*_), such that **s̃**_*p*_^2^ = *t*_*p*_(*t*_*p*_ + 1). *H̃* can be diagonalized independently in each sector. In the following, we focus on a site *p*. As shown in eq 68, we separate *H̃* into a term *H̃*_rest_ that is independent of **s̃**_*p*_, eq 69, and a term **s̃**_*p*_·**B̃** for the interaction of **s̃**_*p*_ with its environment. The superscript *T* in eq 70 denotes matrix transposition.

A37
$$\mathrm{H̃}={\mathrm{H̃}}_{\mathrm{rest}}+\sum_{m>p}{\mathrm{s̃}}_{p}\cdot D_{\mathrm{pm}}\cdot {\mathrm{s̃}}_{m}+\sum_{n<p}{\mathrm{s̃}}_{n}\cdot D_{\mathrm{np}}\cdot {\mathrm{s̃}}_{p}={\mathrm{H̃}}_{\mathrm{rest}}+{\mathrm{s̃}}_{p}\cdot \mathrm{B̃}$$

A38
$${\mathrm{H̃}}_{\mathrm{rest}}=\sum_{\underset{m,n\neq p}{m<n}}{\mathrm{s̃}}_{m}\cdot D_{\mathrm{mn}}\cdot {\mathrm{s̃}}_{n}$$

A39
$$\mathrm{B̃}=\sum_{m>p}D_{\mathrm{pm}}\cdot {\mathrm{s̃}}_{m}+\sum_{n<p}D_{\mathrm{np}}^{T}\cdot {\mathrm{s̃}}_{n}$$

The ground state in a sector (*t*_1_, *t*_2_, ···, *t*_*N*_) is degenerate with respect to the linearly independent ways of coupling the auxiliaries into their respective local spins (*t*_1_, *t*_2_, ···, *t*_*N*_); additional degeneracies may result from non-Abelian symmetries of the physical Hamiltonian *H* (e.g., spin-rotational symmetry in the Heisenberg model) but are of no concern here. Therefore, we may choose—without loss of generality—a ground state |Ψ_*t*_*p*__⟩ that has (*s*_*p*_ – *t*_*p*_) localized singlets at site *p*. We assume that **κ**_*p*,1_ and **κ**_*p*,2_ form a singlet if *s*_*p*_ – *t*_*p*_ ≥ 1, **κ**_*p*,3_ and **κ**_*p*,4_ form a second singlet if *s*_*p*_ – *t*_*p*_ ≥ 2, etc. The remaining 2*t*_*p*_ qubits are coupled into a total spin *t*_*p*_. Thus, |Ψ_*t*_*p*__⟩ comprises a product of singlets at *p*, see eq 71

A40
$$\left|\Psi_{t_{p}}\right\rangle ={\left|0\right\rangle }_{\left(1,2\right)}{\left|0\right\rangle }_{\left(3,4\right)}{\left|0\right\rangle }_{\left(5,6\right)}\cdot \cdot \cdot \left|\psi_{t_{p}}\right\rangle$$

where |*ψ*_*t*_*p*__⟩ represents the remaining 2*t*_*p*_ auxiliaries as well as the other sites (in general, these 2*t*_*p*_ qubits are entangled with the other sites). When excluding a pair (1,2) from the product, we use the notation of eq 72

$${|}_{\left(1,2\right)}\Psi_{t_{p}}\rangle \equiv {\left|0\right\rangle }_{\left(3,4\right)}{\left|0\right\rangle }_{\left(5,6\right)}\cdot \cdot \cdot \left|\psi_{t_{p}}\right\rangle$$
A41

We shall prove the maximal local-spin conjecture inductively. In the basis step, we consider a sector with minimal spin at *p*, i.e., (*t*_*p*_)_min_ = 0 or ${\left(t_{p}\right)}_{\min}=\frac{1}{2}$ , depending on whether 2*s*_*p*_ is even or odd, respectively. The other values {*t*_*i*_}, *i* ≠ *p*, remain fixed throughout. In the following, we denote (*t*_*p*_)_min_ by *t*_min_ and *t*_*p*_ by *t*. Our aim is to show that the ground-state energy *E*_0_^*t*_min_^ ≡ ⟨Ψ_*t*_min__|*H̃*|Ψ_*t*_min__⟩ is an upper bound to the ground state *E*_0_^*t*_min_+1^ of the respective sector where the spin at *p* is increased by one unit

A42
$$E_{0}^{t_{\min}+1}\leq E_{0}^{t_{\min}}$$

By the variational principle, eq 73 holds if there exists a trial state |Φ_*t*_min_+1_⟩ with local spin (*t*_min_ + 1) that satisfies *E*_trial_ ≤ *E*_0_^*t*_min_^. In the following, we transform |Ψ_*t*_min__⟩ into |Φ_*t*_min_+1_⟩ and show that the supposed inequality holds. To this end, the singlet |0⟩ formed by **κ**_*p*,1_ and **κ**_*p*,2_ is first decoupled into a triplet |1⟩ through a unitary operator *R*, i.e., *R*|0⟩ = |1⟩. A general triplet state is a superposition of three possible projections on a spatial direction defined by a unit vector **u**, i.e., |1, −1⟩_**u**_, |1, 0⟩_**u**_, and |1, +1⟩_**u**_. A particular instance of *R* is *R*_**u**_, which releases |0⟩ into |1, −1⟩_**u**_, see eqs 74 and 75

A43
$$R_{u}\left|0\right\rangle ={\left|1,-1\right\rangle }_{u}$$

A44
⟨1,−1|u(κp,1+κp,2)|1,−1⟩u=−u

A normalized trial state |θ_*t*_⟩ is constructed from |Ψ_*t*_⟩ by releasing a singlet pair (*a*,*b*)­

$$\left|\theta_{t}\right\rangle \equiv R_{\left(a,b\right),u}\left|\Psi_{t}\right\rangle ={\left|1,-1\right\rangle }_{\left(a,b\right),u}{|}_{\left(a,b\right)}\Psi_{t}\rangle$$
A45

Using eq 75 and the definition of eq 76, *E*_trial_ is evaluated in eq 77

A46
Etrial=⟨θtmin|H̃|θtmin⟩=⟨Ψtmin|H̃|Ψtmin⟩+⟨1,−1|u(κp,1+κp,2)|1,−1⟩u·⟨Ψtmin|B̃|Ψtmin⟩=E0tmin−u·⟨B̃⟩

where ⟨**B̃**⟩ ≡ ⟨Ψ_*t*_min__|**B̃**|Ψ_*t*_min__⟩. A direction **u** can always be chosen such that *E*_trial_ ≤ *E*_0_^*t*_min_^, and, unless ⟨**B̃**⟩ = 0, a strict inequality can be obtained, *E*_trial_ < *E*_0_^*t*_min_^. If *t*_min_ = 0, then |θ_*t*_min__⟩ has a definite *t*_*p*_ = 1, and the induction basis has been established.

On the other hand, if $t_{\min}=\frac{1}{2}$ , then |θ_*t*_min__⟩ is a superposition of $t=\frac{1}{2}$ and $t=\frac{3}{2}$ states. To analyze this case, consider that any state |Ξ⟩ can be expressed as a linear combination of normalized states |Ξ_*t*_⟩ with definite on-site spin at *p*, see eq 78

A47
$$\left|\Xi \right\rangle =\sum_{t}c_{t}\left|\Xi_{t}\right\rangle$$

The individual contributions are picked out by a (Hermitian and idempotent) spin-projection operator *P* ^(*t*)^, eq 79

A48
$$P^{\left(t\right)}\left|\Xi \right\rangle =c_{t}\left|\Xi_{t}\right\rangle$$

Note that the projector can be expressed in the Löwdin^45^ form of eq 80

A49
$$P^{\left(t\right)}=\prod_{l\neq t}\frac{{\mathrm{s̃}}_{p}^{2}-l\left(l+1\right)}{t\left(t+1\right)-l\left(l+1\right)}$$

but such a specific expression is not needed here. For a Hamiltonian with local spin as a good quantum number, [*H̃*, **s̃**_*p*_^2^] = 0 (thereby [*H̃*, *P* ^(*t*)^] = 0), the energy is the probability-weighted sum of eq 81

A50
$$E=\left\langle \Xi \right|\mathrm{H̃}\left|\Xi \right\rangle =\sum_{t}{\left|c_{t}\right|}^{2}\left\langle \Xi_{t}\right|\mathrm{H̃}\left|\Xi_{t}\right\rangle =\sum_{t}{\left|c_{t}\right|}^{2}E_{t}$$

In the specific case of |θ_*t*_min__⟩ with $t_{\min}=\frac{1}{2}$ , eq 77 yields *E*_trial_ = |*c*_1/2_|^2^ *E*_1/2_ + |*c*_3/2_|^2^ *E*_3/2_, with |*c*_1/2_|^2^ + |*c*_3/2_|^2^ = 1. But as *E*_0_^*t*_min_^ ≤ *E*_1/2_, we obtain *E*_3/2_ ≤ *E*_0_^*t*_min_^ for the trial state |Φ_*t*_min_+1_⟩ = *P* ^(3/2)^|θ_*t*_min__⟩.

In summary, in both cases, *t*_min_ = 0 or $t_{\min}=\frac{1}{2}$ , projection |Φ_*t*_min_+1_⟩ ≡ *P* ^(*t*_min_+1)^|θ_*t*_min__⟩ yields *E*_*t*_min_+1_ ≤ *E*_0_^*t*_min_^ (or E_*t*_min_+1_ < *E*_0_^*t*_min_^ if ⟨**B̃** ≠ 0⟩). Thus, by the variational principle, *E*_0_^*t*_min_+1^ ≤ *E*_trial_, which proves that increasing the spin at *p* from *t*_min_ to (*t*_min_ + 1) does not increase the ground-state energy.

We proceed inductively and consider a ground state |Ψ_*t*_⟩ in a sector with *t*_min_ < *t* < *s*_*p*_. Like in the previous derivation, a singlet (*a*,*b*) in |Ψ_*t*_⟩ can be decoupled to yield a favorable interaction between the triplet and the environment, cf. eq 82, where ⟨**B̃**⟩ = ⟨Ψ_*t*_|**B̃**|Ψ_*t*_⟩. The choice of a suitable **u** for |θ_*t*_⟩ = *R*_(*a*,*b*),**u**_|Ψ_*t*_⟩ does not depend on the specific pair (*a*,*b*).

A51
$$E_{\mathrm{trial}}=\left\langle \theta_{t}\right|\mathrm{H̃}\left|\theta_{t}\right\rangle =E_{0}^{t}-u\cdot \left\langle \mathrm{B̃}\right\rangle \leq E_{0}^{t}$$

Now, |θ_*t*_⟩ is a superposition of (*t* – 1), *t*, and (*t* + 1) states, eq 83

A52
$$E_{\mathrm{trial}}=\left\langle \theta_{t}\right|\mathrm{H̃}\left|\theta_{t}\right\rangle ={\left|c_{t-1}\right|}^{2}E_{t-1}+{\left|c_{t}\right|}^{2}E_{t}+{\left|c_{t+1}\right|}^{2}E_{t+1}$$

with |*c*_*t* – 1_|^2^ + |*c*_*t*_|^2^ + |*c*_*t*+1_|^2^ = 1. Inducing from the base case yields the first inequality in [Disp-formula eq84]

A53
$$E_{\mathrm{trial}}\geq {\left|c_{t-1}\right|}^{2}E_{0}^{t}+{\left|c_{t}\right|}^{2}E_{t}+{\left|c_{t+1}\right|}^{2}E_{t+1}\geq \left({\left|c_{t-1}\right|}^{2}+{\left|c_{t}\right|}^{2}\right)E_{0}^{t}+{\left|c_{t+1}\right|}^{2}E_{t+1}$$

and combining eqs 82 and 84 yields eq 85

A54
$$E_{0}^{t}\geq \left({\left|c_{t-1}\right|}^{2}+{\left|c_{t}\right|}^{2}\right)E_{0}^{t}+{\left|c_{t+1}\right|}^{2}E_{t+1}\Leftrightarrow E_{0}^{t}\geq E_{t+1}$$

Thus, the trial state |Φ_*t*+1_⟩ ≡ *P* ^(*t*+1)^|θ_*t*_⟩ does not have a higher energy than |Ψ_*t*_⟩, so that *E*_0_^*t*+1^ ≤ *E*_0_^*t*^. This proves the monotonicity with respect to increasing the on-site spin at *p*. Iterating over the sites then affords the result that all on-site spins are maximal in the ground state.

If the Hamiltonian conserves *S*_*z*_, then a ground state |Ψ_*t*_^*M*^⟩ in a magnetization sector *M* can be similarly used to construct a trial state |Ψ_*t*+1_^*M*^⟩, which remains in the same *M*-sector when choosing a projection of 0 of the triplet with respect to the *z*-axis (**u** = **z**). Although such a triplet state does not interact with the environment **B̃**, we can still conclude that the ground state can be chosen to have maximal local spins in each *M*-sector.

Although not relevant for the present work, we may speculate that the maximal local-spin principle might more generally apply to the ground state in each symmetry sector Γ of a bilinear Hamiltonian, e.g., in each total-spin and magnetization sector (*S*,*M*) or for combinations of total-spin and point-group symmetry in the isotropic Heisenberg model,^46–49^ or for symmetry groups comprising combined spin rotations and spin permutations in anisotropic systems with spatial symmetry.^50,51^ Besides, we have not been concerned with identifying the most general class of spin Hamiltonians (beyond bilinear) where the maximal local-spin property may hold.
